# Weighted Gene Co-expression Network Analysis Identifies FKBP11 as a Key Regulator in Acute Aortic Dissection through a NF-kB Dependent Pathway

**DOI:** 10.3389/fphys.2017.01010

**Published:** 2017-12-04

**Authors:** Tao Wang, Xingwei He, Xintian Liu, Yujian Liu, Wenjun Zhang, Qiang Huang, Wanjun Liu, Luyang Xiong, Rong Tan, Hongjie Wang, Hesong Zeng

**Affiliations:** ^1^Division of Cardiology, Department of Internal Medicine, Tongji Hospital, Tongji Medical College, Huazhong University of Science and Technology, Wuhan, China; ^2^Department of Cardiology, Wuhan Asia Heart Hospital, Wuhan, China; ^3^Divison of Cardiology, the Fifth Hospital of Wuhan, Wuhan, China

**Keywords:** WGCNA, FKBP11, NF-kB p65 subunit, MMPs, pro-inflammatory cytokines, vascular inflammation, macrophage transmigration, acute aortic dissection

## Abstract

Acute aortic dissection (AAD) is a life-threatening disease. Despite the higher risk of mortality, currently there are no effective therapies that can ameliorate AAD development or progression. Identification of meaningful clusters of co-expressed genes or representative biomarkers for AAD may help to identify new pathomechanisms and foster development of new therapies. To this end, we performed a weighted gene co-expression network analysis (WGCNA) and calculated module-trait correlations based on a public microarray dataset (GSE 52093) and discovered 9 modules were found to be related to AAD. The module which has the strongest positive correlation with AAD was further analyzed and the top 10 hub genes *SLC20A1, GINS2, CNN1, FAM198B, MAD2L2, UBE2T, FKBP11, SLMAP, CCDC34*, and *GALK1* were identified. Furthermore, we validated the data by qRT-PCR in an independent sample set originated from our study center. Overall, the qRT-PCR results were consistent with the results of the microarray analysis. Intriguingly, the highest change was found for *FKBP11*, a protein belongs to the FKBP family of peptidyl-prolyl cis/trans isomerases, which catalyze the folding of proline-containing polypeptides. In congruent with the gene expression analysis, FKBP11 expression was induced in cultured endothelial cells by angiotensin II treatment and endothelium of the dissected aorta. More importantly we show that FKBP11 provokes inflammation in endothelial cells by interacting with NF-kB p65 subunit, resulting in pro-inflammatory cytokines production. Accordingly, siRNA mediated knockdown of FKBP11 in cultured endothelial cells suppressed angiotensin II induced monocyte transmigration through the endothelial monolayer. Based on these data, we hypothesize that pro-inflammatory cytokines elicited by FKBP11 overexpression in the endothelium under AAD condition could facilitate transendothelial migration of the circulating monocytes into the aorta, where they differentiate into active macrophages and secrete MMPs and other extracellular matrix (ECM) degrading proteins, contributing to sustained inflammation and AAD. Taken together, our data identify important role of FKBP11 which can serve as biomarker and/or therapeutic target for AAD.

## Introduction

Aortic dissection, which damages the integrity of the arterial wall, is a life-threatening disease. An acute aortic dissection (AAD) was defined when the process is less than 14 days. Patients with diagnosed AAD have a high mortality both before and after hospital admission (Meszaros et al., 2000; Olsson et al., 2006; Steuer et al., 2013).

Despite the higher risk of mortality associated with AAD, there are no effective drug therapies that have been shown to restrict AAD development or progression. Currently, open or endovascular surgical repair remains the major form of treatment (Zhang et al., 2016). Therefore, the elucidation of the cellular and molecular basis for this disease is urgently required for the design of novel pharmacologic therapies and management strategies.

At present, the systems biology analysis of gene expression profiling largely boosters the scientific investigation, it can be a powerful tool to uncover the possible pathomechanisms of AAD from the perspective of gene regulation. Weighted gene co-expression network analysis (WGCNA) is a systems biology method for describing the correlation patterns among genes across microarray samples (Zhao et al., 2010). WGCNA can be used for finding clusters (modules) of highly correlated genes, for summarizing such clusters using the module eigengene or an intramodular hub gene, for relating modules to one another and to external sample traits, and for calculating module membership measures. Correlation networks facilitate network based gene screening methods that can be used to identify candidate biomarkers or therapeutic targets. These methods have been widely used in various biological aspects, e.g., cancer, mouse genetics, analysis of acute myocardial infarction, etc (Langfelder and Horvath, 2008; Chen et al., 2016; Liu W. et al., 2017).

To this end, within the current study, we constructed correlation networks using gene expression data from publicly accessible resources. We conducted GO and pathway enrichment analyses and gene module alteration in patients with AAD, and furthermore, hub genes were recognized, which could be used as biomarkers and potential therapeutic targets for AAD. The top 10 hub genes were subsequently verified by qRT-PCR in an independent sample set originated from our study center and the most relevant gene *FKBP11* was selected for further functional assays *in vitro* and *ex vivo*.

## Materials and methods

### Materials

The following antibodies were used in the current study: mouse monoclonal antibodies: CD31 (Abcam), Mac-2 (Santacruz, Germany), α-SMA (Boster, Wuhan, China); rabbit polyclonal antibodies: FKBP11 (Bioss, Beijing, China), p65, p-p65 (Cusabio, Wuhan, China), and MMP-9 (Santacruz, Heidelberg, Germany); VCAM-1, ICAM-1, MCP-1 (Wanleibio, Shenyang, China); β-actin (New England Biolabs, Germany); IL1-β (Sigma-Aldrich, Taufkirchen, Germany). The following HRP conjugated secondary antibodies were used for immunoblotting: rabbit IgG (New England Biolabs, Germany), mouse IgG (Abcam). The following secondary antibodies for immunofluorescence were used: Texas red conjugated anti-rabbit and FITC conjugated anti-mouse (Vector Laboratories, CA, USA).

The following reagents used in the current study were: Ang II (Sigma-Aldrich, St. Louis, MO), Trypsin-EDTA and HEPES (Gibco, Germany), fetal bovine serum (Sigma-Aldrich, St. Louis, MO), Protein A-Agarose Immunoprecipitation Reagent (Santacruz, Germany); phosphatase inhibitor cocktail (Roche Applied Science, Penzberg, Germany) and protease inhibitor cocktail (Roche diagnostics GmbH, Mannheim, Germany); BCA reagent (Perbio Science, Bonn, Germany); Vectashield mounting medium with DAPI (Vector Laboratories, CA, USA); PVDF membrane and immobilion enhanced chemiluminescence reagent (Millipore GmbH, Germany).

### Ethics statement and sample collection

All studies were conducted under protocols approved by the Ethics Committee of Tongji medical college, Huazhong University of Science and Technology (Wuhan, China). Written informed consent was obtained from the patients or their relatives in accordance with the Declaration of Helsinki. Six consecutive patients (3 males and 3 females, mean age 48.0 ± 9.8 years) underwent emergency surgery for Stanford type A AAD were recruited. Acute aortic dissection is defined as dissection detected within 2 weeks of the onset of symptoms (Steuer et al., 2013). Patients with Marfan syndrome, Ehlers-Danlos syndrome, and other known connective tissue disorders were excluded. None of the patients had bicuspid aortic valve, aortic coarctation, Takayasu's arteritis, coronary artery disease, or cocaine use. Dissected ascending aorta specimens were obtained through operation, during which the tear site was identified and a strip of aortic wall including the tear site was carefully excised. Any adherent mural thrombus was removed from the aortic wall. Each sample was divided into three parts. One part was used for histological assessment, one part for RNA isolation and the rest for protein extraction. As control samples, normal ascending aorta specimens were collected from 6 organ donors (mean age 42.6 ± 8.3 years). Normal control samples were treated in the exactly same manner as the AAD samples (Pan et al., 2014). All subjects were of Asian origin. Detailed clinical information of the individuals enrolled in the study is shown in Table 1. There are no statistically significant differences of the demographics between the two groups.

**Table 1:** Characteristics of patients and controls.

**Parameter**	**Controls**	**Patients**
	**(*n* = 6)**	**(*n* = 6)**
Age (years)	42.6 ± 8.3	48.0 ± 9.8
Men (%)	4 (66.7)	3 (50)
Smoking (%)	3 (50)	2 (33.3)
Hypertension (%)	1 (16.7)	5 (83.3)
Diabetes Mellitus (%)	0 (0)	0 (0)
Connective tissue disorders (%)	0 (0)	0 (0)
Hereditary vascular disease (%)	0 (0)	0 (0)
Takayasu's arteritis (%)	0 (0)	0 (0)
Cocaine use (%)	0 (0)	0 (0)
Deceleration trauma (%)	0 (0)	0 (0)
Iatrogenic maneuver (%)	0 (0)	0 (0)
Coronary artery disease (%)	0 (0)	0 (0)
Chronic obstructive disease (%)	0 (0)	0 (0)

*Data are presented as mean ± SEM or number (%)*.

### Total RNA isolation and quantitative real-time polymerase chain reaction

According to the microarray results, the top 10 hub genes were chosen for further validation by qRT-PCR in AAD patients vs. healthy controls. The qRT-PCR was performed as described elsewhere (Liu J. et al., 2017). Aorta tissue samples were collected and pulverized under liquid nitrogen, total RNA was extracted with Trizol reagent (TaKaRa, Japan). Then, 1 μg RNA was added to a 20 μL reaction volume for cDNA reverse transcription using the Prime Script™ RT reagent Kit with gDNA Eraser (TaKaRa, Japan), and quantitative real-time polymerase chain reaction (qRT-PCR) was performed using the SYBR® Premix Ex Taq™ (TaKaRa, Japan) in a Step One Plus real-time PCR system (Applied Biosystems, USA). Gene expression was quantified according to the 2^−ΔΔCt^ method. Primer sets for selected genes were designed by TianYi Huiyuan (Wuhan, China) and their sequences are available in Table 2. The expression data were normalized to the reference 18s rRNA.

**Table 2:** PCR primers for quantitative real-time PCR.

**Gene**	**Primer sequence (5**′→**3**′**)**	
SLC20A1	F: GTGAACAGAAGGGCGAAGA	R: CGAATGACCCAAAGCAGG
GINS2	F: ATCTACCTCATCGGGGGGGA	R: AGGGAGCAGGCGACATTTC
CNN1	F: AGCCCCACGACATTTTTG	R: GTTTTTACAGCACCCCGA
FAM198B	F: TACCCAAGCCTGAATCGG	R: TCACTCCTGTCAAAGAAACCC
MAD2L2	F: ATGTGCTCTGCGAGTTCCT	R: TGGGCGGTGCTCTTTATCC
UBE2T	F: GATTCTGCTGGAAGGATTTGT	R: GGGTCATCAGGGTTGGGTT
FKBP11	F: AGACACGCTTCACATACACTACA	R: TCGCTTCTCTCCCACACAC
SLMAP	F: CAGATGGTATGGAAGCCCG	R: AAAGCCTGCCAACTGGTATC
CCDC34	F: CAGTTTGACGAGGACGACG	R: TCTGATTCAACCTGAGTGCTG
GALK1	F: GGAACACACGGACTACAACCA	R: AGGAGAGACACCAGCCCATC
18SrRNA	F: GTAACCCGTTGAACCCCATT	R: CCATCCAATCGGTAGTAGCG

### Microarray data and analysis of differentially-expressed genes

The microarray data sets GSE 52093 was obtained from the NCBI Gene Expression Omnibus (GEO) (http://www.ncbi.nlm.nih.gov/geo) database, which was performed in seven human type A AAD samples and five control samples, and the platforms was Illumina HumanHT-12 V4.0 expression beadchip (Illumina Inc., San Diego, CA, USA) (Pan et al., 2014). For multiple probes corresponding to one gene, there average expression value was taken as the gene expression value. After that, gene expression values were normalized using preprocessCore package (version 1.28.0, http://www.bioconductor.org/package/release/bioc/html/preprocessCore.html) (Bolstad et al., 2003), and were performed with log2 transformation. Only probes that were annotated to an Entrez gene ID were kept for further analysis. Expression profiles of probes mapping to the same Entrez gene ID were averaged.

Linear models for microarray data (Limma) package (version1.22.0, http://www.bioconductor.org/packages/release/bioc/html/limma.html) in R was utilized to identify the Differentially-Expressed Genes (DEGs) between type A AD and control samples. Genes with fold change >2 and false discovery rate (FDR) < 0.05 were selected for subsequent analysis (Smyth, 2004).

### GO and pathway enrichment analyses

Functional enrichment of GO and KEGG pathways analyses were performed with the DAVID Bioinformatics Tool (https://david.ncifcrf.gov/, version 6.8) (Huang da et al., 2009a,b). *P*-value < 0.05 was considered to be significant enrichment. Functional enrichment analysis of the network module genes was also conducted according to the above methods.

#### Construction of weighted gene co-expression network and identification of significant modules

WGCNA was used to cluster groups of strongly co-expressed genes into co-expression networks among DEGs (Zhang and Horvath, 2005; Langfelder and Horvath, 2008). In data processing, the genome-wide gene expression data was preliminarily filtered, followed by measuring the consistency of gene expression profiles by Pearson correlation, and finally using the power adjacent function to Pearson correlation matrix, data was transformed into weighted gene co-expression networks (Chen et al., 2016).

Network module is a cluster of closely interconnected genes. During module selection, the adjacency matrix, a measure of topology similarity, is transformed into the topological overlap matrix (TOM), and modules are detected by cluster analysis (Yip and Horvath, 2007). To determine whether the modules were associated with aortic AAD, we then analyzed the importance of genes by the *t*-test.

### Identification of hub genes

The hub genes of modules, described as the most closely associated with disease, often have more biological significance compared with the hub genes of global networks (Goh et al., 2007). A gene can be considered as a hub gene if it has a unique character, such as high gene significance (GS), high module membership (MM), and high intramodular connectivity (IC) in the network (Zhang and Horvath, 2005). GS showed the different IC and MM described the significance of genes in various networks. We therefore identified the hub genes in modules through the GS and MM.

### Protein-protein interaction (PPI) network construction and analysis

PPI assessment was employed to infer the interactions among proteins in the selected WGCNA modules with STRING database (https://string-db.org/) (Szklarczyk et al., 2017).

#### Histology and immunohistochemistry

Histological analysis was performed as previously described (Liu J. et al., 2017). Briefly, aorta tissue samples were fixed in 10% neutral-buffered formalin, embedded in paraffin, and cut into sections of 5 μm thickness. Slides were then deparaffinized with xylene, dehydrated with graded ethanol, and elastica van Gieson (EVG) staining was conducted to evaluate the condition of elastic fiber (Ohtani et al., 2004).

For immunohistochemical probing, aorta slides were initially boiled in a pressure cooker containing a citric acid buffer (pH 6.0) for 20 min to retrieve antigens. Then the slides were blocked with 5% BSA for 1 h and incubated with primary antibodies overnight at 4?, and with corresponding secondary antibody for 30 min at room temperature. Immunolabels were detected with 3, 3-Diaminobenzidine (DAB), after which the nuclei were counterstained with hematoxylin. Slides were inspected under an optical microscope at 400 × magnification. For quantification analysis, the percentage of positive area for immunohistochemistry was determined using ImageJ software (Madhusudhan et al., 2015).

#### Double immunofluorescence staining

Colocalization studies were performed with double immunofluorescence staining methods. The sections were prepared as described in histological analysis till the incubation of secondary antibodies. Corresponding fluorescently labeled secondary antibodies (anti-mouse IgG FITC: 1:200, anti-rabbit IgG-Texas red: 1:200) were added for 60 min and sections were rinsed twice in PBS. Slides were covered with vectashield mounting medium containing nuclear stain DAPI (Vector Laboratories, CA, USA). Specimens were analyzed on a Leica SP5 confocal microscope (Madhusudhan et al., 2012).

### Cell culture and siRNA transfection

THP-1 cells, a pro-monocytic cell line (ATCC® TIB-202™), were cultured in RPMI 1640 (Gibco, CA,USA) supplemented with 10% fetal bovine serum (Sigma-Aldrich, St. Louis, MO), 10 mM HEPEs, 0.1 mM MEM non-essential amino acids, 1 mM sodium pyruvate, and 100 nM penicillin/streptomycin (Life Technologies), were maintained at 5% CO_2_, 37°C. EA.hy926 cells were obtained from ATCC (Manassas, VA) and cultured in RPMI 1640 medium (Gibco, CA, USA) with 10% fetal bovine serum (Sigma-Aldrich, St. Louis, MO) in a humidified atmosphere of 5% CO_2_. Primary human vascular endothelial cells (HUVECs, Cat No.: CP-H082, Procell Life Science&Technology Co,.Ltd., Wuhan, China)were cultured in endothelial culture medium (Cat No.: CM-H084, Procell Life Science&Technology Co,.Ltd., Wuhan, China). Specific siRNAs targeting FKBP11 purchased from Ruibo (Guangzhou, China), and transfected into cells with TurboFect Transfection Reagent (Thermo Fisher Scientific, Waltham, MA USA) according to the manufacturer's protocol. The sequence of FKBP11-siRNA was listed as following: FKBP11-Si1, GGGCAATCATTCCTTCTCA; FKBP11-Si2, GAGAAGCGAAGGGCAATCA; FKBP11-Si3, CTCACTTGGCCTATGGAAA. The scrambled siRNA controls were used as a negative control.

### Cytofluorescence

Immunofluorescence analyses of immortalized human endothelial cell line EA.hy926 cells were done on collagen-coated coverslips. EA.hy926 cells were fixed in 3.7% paraformaldehyde (PFA) and washed three times with PBS containing 0.2% Trition X-100. Coverslips were blocked with blocking reagent for 1 h and incubated overnight at 4°C with a primary antibody against p-p65. Coverslips were washed three times with PBS and corresponding fluorescently labeled secondary antibodies were added for 60 min. Coverslips were rinsed thrice in PBS and placed on vectashield mounting medium containing nuclear stain DAPI. Specimens were analyzed on a Leica SP5 confocal microscope. Nuclear localization of p-p65 was analyzed using an ImageJ-Fiji (http://imagej.nih.gov) macro including automated detection of nuclei in the DAPI channel and comparison of p-p65 levels within the nucleus vs. within a band mask surrounding the nucleus. DAPI stained and fluorescently labeled images were acquired individually (Madhusudhan et al., 2015).

### Western blotting

Proteins were collected from the aortic tissues or cultured endothelial cells using RIPA lysis buffer. Protein concentration was quantified using BCA reagent. Equal amounts of protein were electrophoretically separated on 10% SDS polyacrylamide gel, transferred to PVDF membranes and probed with desired primary antibodies overnight at 4°C. Membranes were then washed with TBST and incubated with anti-mouse IgG (1:5,000) or anti-rabbit IgG (1:2,000) horseradish peroxidase conjugated antibodies as indicated. Blots were developed with the enhanced chemiluminescence system. To compare and quantify levels of proteins, the density of each band was measured using Image J software. Equal loading for total cell or tissue lysates was determined by β-actin western blot (Dong et al., 2015; Madhusudhan et al., 2015).

### Co-immunoprecipitation

Immunoprecipitation of total cellular proteins from endothelial cells was done as described elsewhere (Shahzad et al., 2016) (Dong et al., 2015). In brief, total cellular proteins were extracted with protein lysate containing phosphatase inhibitor and complete protease inhibitor cocktail. Leave part of the centrifuged protein supernatant for input. Lysates were combined with 3 μg of specific antibody and incubated for 2 h at 4°C. Immunoprecipitates were purified with protein A/G agarose beads and washed with PBS containing phosphatase inhibitor and protease inhibitor cocktail. Immunoprecipitates were fractionated by 10% SDS-PAGE, transferred to membranes, and subjected to immunoblotting with appropriate primary and secondary antibodies as described above.

### *In vitro* transmigration assay

Transmigration assay was performed as previously described (Seehaus et al., 2009). In brief, Corning® Transwell® polycarbonate membrane inserts (6.5 mm diameter, 8 μm pore size) were used. Endothelial monolayer was incubated with Ang II 24 h after transfected with FKBP11-Si RNA or NC-Si RNA for 24 h. Then 5 × 10^5^ THP-1 cells were seeded into each upper compartment containing 100 μL serum-free RPMI medium. In preincubation experiments cells were washed twice before THP-1 cells were added to the endothelial cell monolayer. Following incubation at 37°C in a humidified 5% CO_2_ incubator for 12 h the upper compartments were removed and THP-1 cells transmigrated into the lower compartment were counted after stained with crystal violet. Each assay was performed in triplicates and repeated at least three times. Cells were counted in five randomly selected squares per well and presented as the number of migrated cells/field.

### Statistical analysis

One-way ANOVA and the Student's *t*-test were used to evaluate any variations in outcomes between different groups by SPSS 19.0 statistical software. Most bioinformatics analyses including WGCNA were performed in R (version 3.3.1) with default test statistics and cutoff values as specified in individual method sections. *P*-value < 0.05 was considered as statistically significant. Results are expressed as mean ± SEM. A *P*-value < 0.05 was considered as statistically significant.

## Results

### DEGs selection and hierarchical clustering analysis

**Figure 1:**
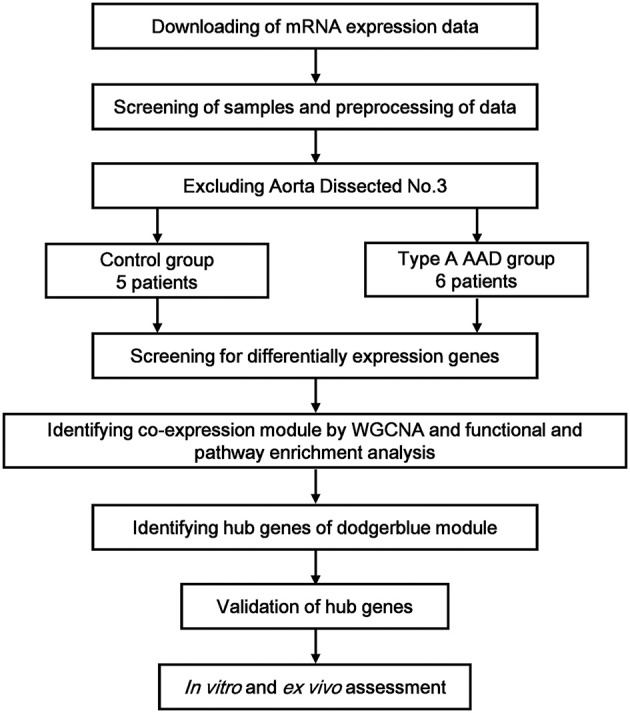
Flow diagram of the study approach. mRNA microarray analyses were performed on aortic specimens obtained from seven AAD patients and five control group (GSE 52093). After excluding Aorta Dissected No. 3 through clustering analysis, six human type A AAD samples and five control samples were included in the subsequent analysis. From among differentially-expressed genes, network analysis of gene expression in aortic dissection identifies 9 modules of co-expression genes. Afterwards, the relevance between each module and aortic dissection were tested through calculating the relevance between the feature vectors of modules and phenotypes, and the dodgerblue module was identified as the most relevant module. Functional and pathway enrichment analysis were performed for the genes in the dodgerblue module. The top 10 genes with the highest connectivity in dodgerblue module were taken as hub genes. Validation quantitative polymerase chain reaction (qRT-PCR) was performed for the 10 hub genes in samples obtained from our institute, including 6 organ donors and 6 AAD patients. At last, EA.hy926 cells and THP-1 cells were used for functional verification *in vitro*.

We used R software and corrected batch effect by Bayesian methods, then removed the probes without corresponding annotation information. To determine the expression values of each gene, we used multiple GSE52093 probes corresponding to the median expression value of that gene. After excluding Aorta Dissected No.3 through clustering analysis (Supplementary Figure 1), six human type A AAD samples and five control samples were included in the subsequent analysis (Figure 1).

**Figure 2:**
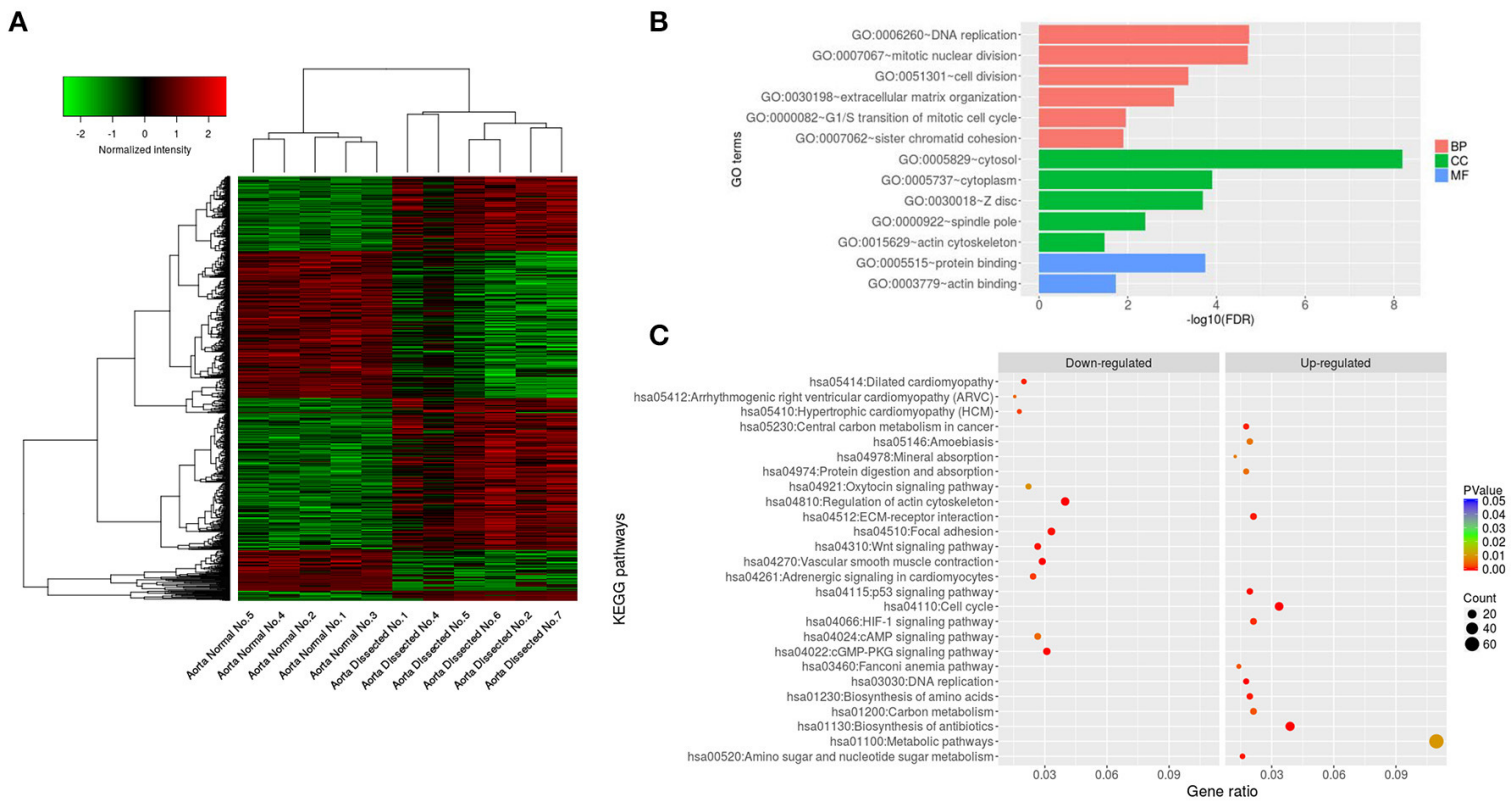
Hierarchical clustering analysis, GO and pathway enrichment analysis. **(A)** DEGs can be effectively divided into AAD and control groups. Red indicates that the gene that is upregulated and green represents down-regulated genes. **(B)** The significant GO terms that conformed to a *P* < 0.05 were screened. **(C)** Fisher's exact test was used to select the significant pathway, identified by a *P* < 0.05. GO, Gene ontology; BP, Biological process; CC, Cellular component; MF, Molecular function.

By using algorithms provided in the Limma package, we calculated the data and obtained lists of differentially expressed genes. Hierarchical clustering analysis was obtained for the DEGs from 11 samples of the AAD patients and control groups. The general gene expression patterns were evidently different in the two groups by Tree View (Figure 2A).

### GO enrichment analysis of DEGs and pathway enrichment analysis

To reveal AAD–related biological processes, we conducted a gene ontology (GO) functional enrichment analysis of the DEGs was performed. The majority of GO terms were associated with the biological processes (BP) such as DNA replication, mitotic nuclear division, extracellular matrix organization, G1/S transition of mitotic cell cycle, and sister chromatid cohesion, with cellular component (CC) like cytosol, cytoplasm, Z disc, spindle pole and actin cytoskeleton, and with molecular function (MF) like protein binding and actin binding (Figure 2B and Table 3).

**Table 3:** Gene ontology (GO) enrichment analysis.

**ID**	**Category**	**Term**	**Count**	***P*-value**
BP (biological process)	GO:0006260	DNA replication	29	1.01E-08
	GO:0007067	Mitotic nuclear division	38	1.09E-08
	GO:0051301	Cell division	44	2.34E-07
	GO:0030198	Extracellular matrix organization	30	4.98E-07
	GO:0000082	G1/S transition of mitotic cell cycle	19	6.05E-06
	GO:0007062	Sister chromatid cohesion	19	6.97E-06
CC (cellular component)	GO:0005829	Cytosol	255	4.48E-12
	GO:0005737	Cytoplasm	343	8.46E-08
	GO:0030018	Z disc	23	1.38E-07
	GO:0000922	Spindle pole	20	2.80E-06
	GO:0015629	Actin cytoskeleton	28	2.25E-05
MF (molecular function)	GO:0005515	Protein binding	540	1.13E-07
	GO:0003779	Actin binding	34	1.15E-05

To analyze the significant enrichment of the above mentioned DEGs in pathway terms, we used Fisher's exact test to calculate the significance level of the pathway (*P* < 0.05). We performed pathway annotation of DEGs and obtained DEGs involved in all pathway terms with the help of KEGG databases. The DEGs of AAD were enriched mainly in pathways such as cell cycle, DNA replication, ECM-receptor interaction, regulation of actin cytoskeleton, vascular smooth cell contraction, focal adhesion, and Wnt signaling (Figure 2C and Table 4).

**Table 4:** KEGG enrichment analysis of genes.

**ID**	**Category**	**Term**	**Count**	***P*-value**
KEGG_PATHWAY	hsa04510	Focal adhesion	29	3.73E-05
	hsa04110	Cell cycle	21	4.39E-05
	hsa04512	ECM-receptor interaction	16	1.84E-04
	hsa03030	DNA replication	10	2.09E-04
	hsa01130	Biosynthesis of antibiotics	27	3.83E-04
	hsa05146	Amoebiasis	15	0.0042923
	hsa05230	Central carbon metabolism in cancer	11	0.0045431
	hsa04066	HIF-1 signaling pathway	14	0.0056129
	hsa04115	p53 signaling pathway	11	0.0063347
	hsa00520	Amino sugar and nucleotide sugar metabolism	9	0.0072553
	hsa04810	Regulation of actin cytoskeleton	23	0.0079735
	hsa00330	Arginine and proline metabolism	9	0.0092959

### Network analysis indicates basic gene alteration in type A acute aortic dissection tissues

**Figure 3:**
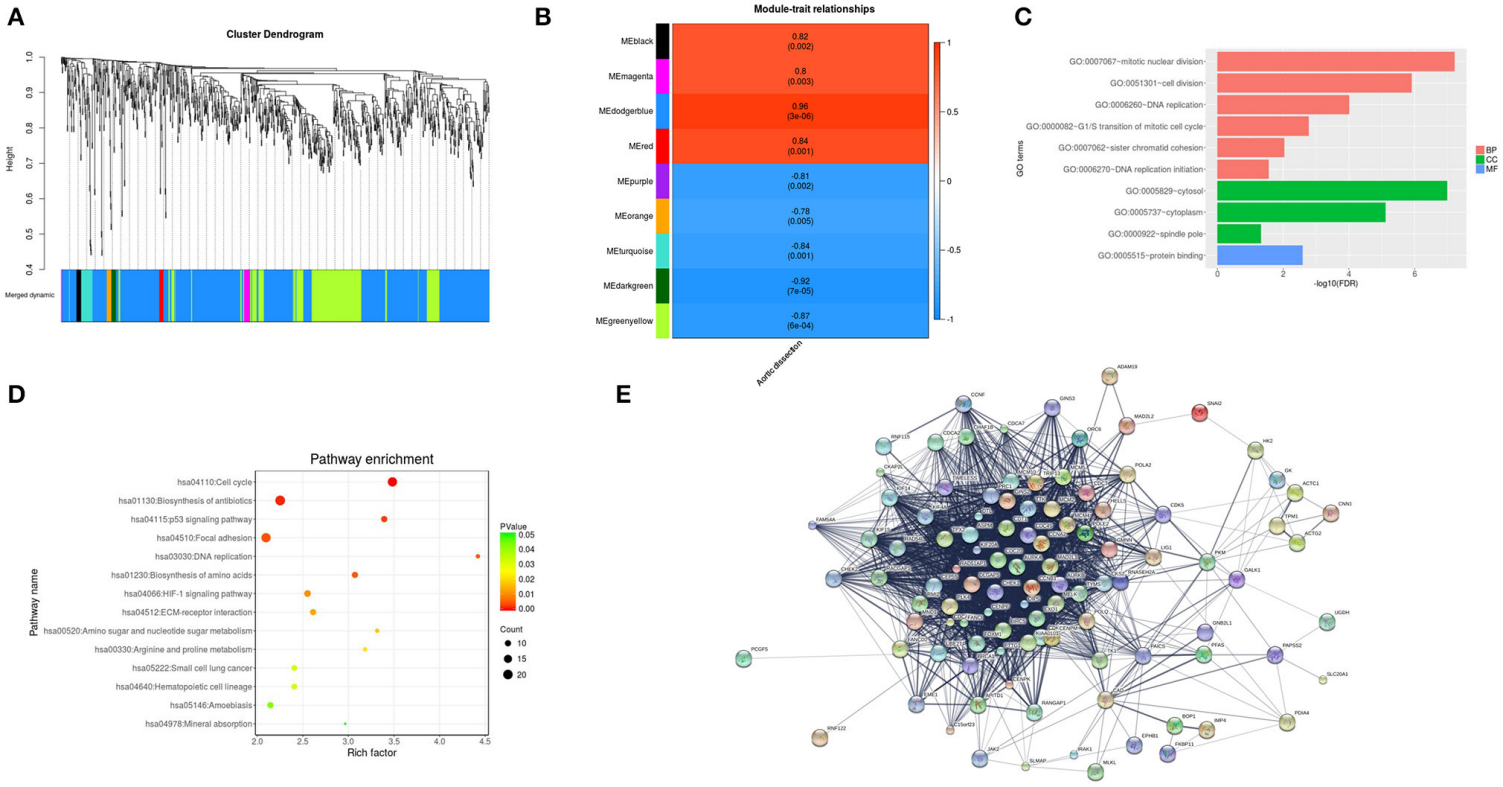
WGCNA analysis and target module and hub genes screening. **(A)** Network analysis of gene expression in AAD identifies distinct modules of co-expression genes. **(B)** Pearson correlation coefficient between the module eigengene of modules and the sample feature vector. Numbers in rectangular represent the correlation coefficients and numbers in brackets indicate the corresponding *P* values. **(C)** The significant GO terms that conformed to a *P* < 0.05 were screened in the dodgerblue module. **(D)** Fisher's exact test was used to select the significant pathway in the dodgerblue module, identified by a *P* < 0.05. **(E)** The constructed PPI networks of the top 10 hub genes in dodgerblue module. GO, Gene ontology; BP, Biological process; CC, Cellular component; MF, Molecular function; PPI, Protein-protein interaction; DEGs, Differentially expressed genes.

WGCNA, which defines transcriptional modules based on Pearson correlation and determines relationship between these modules and different clinical traits, was performed to identify gene co-expression networks associated with AAD clinical-pathological factors. The microarray data Set GSE52093 was used for this analysis. We identified 9 distinct co-expression modules (Figure 3A). The expression data from different genes within each calculated module were used to determine the module eigengenes. Eigengenes were then correlated with external traits to identify modules that are significantly associated with the clinical traits and to find the most significant associations. The calculating module-trait correlation was presented as a heatmap (Figure 3B). We observed that the disease state (AAD vs. Control) showed the most significant correlation with several modules. According to the correlation coefficients, genes clustered in black, magenta, dodgerblue, red modules are positive correlation, while genes in purple, yellow, orange, turquoise, darkgreen, greenyellow modules are negative correlation in AAD tissues.

As shown in Figure 3B, genes clustered in dodgerblue module have the strongest positive correlation with AAD, while genes in darkgreen module have negative correlation with the disease status. To further explore the key regulatory notes, we applied GO and pathway enrichment analysis of the genes in the dodgerblue module (Figure 3B). Similarly, as showed in overall correlation, the most GO terms in dodgerblue module were associated with BP like mitotic nuclear division, cell division, DNA replication, G1/S transition of mitotic cell, and sister chromatid cohesion, CC like cytosol, cytoplasm and spindle pole, and MF like protein binding (Figure 3C). The DEGs of AAD were enriched mainly in cell cycle, DNA replication, ECM-receptor interaction, biosynthesis of amino acid, focal adhesion (Figure 3D). The data indicate that the genes highly significantly associated with the patients' disease state (AAD) are also the most important elements of this module.

A major goal of our research was to analyze the degree of correlation between genes and disease, and to ascertain the significance of genes in the corresponding modules. Then a PPI (protein protein interaction) map was constructed based on the DEGs in the dodgerblue module (Supplementary Figure 2). Network of DEGs in dodgerblue module was comprised of 706 genes, including the top 10 hub genes *SLC20A1, GINS2, CNN1, FAM198B, MAD2L2, UBE2T, FKBP11, SLMAP, CCDC34*, and *GALK1* (Table 5) with the interactived genes of 1, 66, 3, 0, 6, 53, 3, 7, 0, and 11, respectively (Figure 3E).

**Table 5:** Top 10 hub genes identified in PPI network for DEGs.

**Rank**	**ID**	**Name**	**logFC**	***P*-value**
1	SLC20A1	Solute carrier family 20 member 1	1.70	3.75E-07
2	GINS2	GINS complex subunit 2	4.58	6.96E-07
3	CNN1	Calponin 1	−3.70	7.75E-06
4	FAM198B	Family with sequence similarity 198 member B	−1.37	6.35E-06
5	MAD2L2	Mitotic arrest deficient 2 like 2	1.41	5.78E-05
6	UBE2T	Ubiquitin conjugating enzyme E2 T	2.81	1.35E-05
7	FKBP11	FK506 binding protein 11	2.03	2.32E-05
8	SLMAP	Sarcolemma associated protein	−2.54	3.80E-06
9	CCDC34	Coiled-coil domain containing 34	2.37	2.36E-06
10	GALK1	Galactokinase 1	1.12	0.000121

*PPI, Protein–protein interactions; DEGs, Differentially expressed genes; FC, Fold change*.

### Validation of microarrays with qRT-PCR

**Figure 4:**
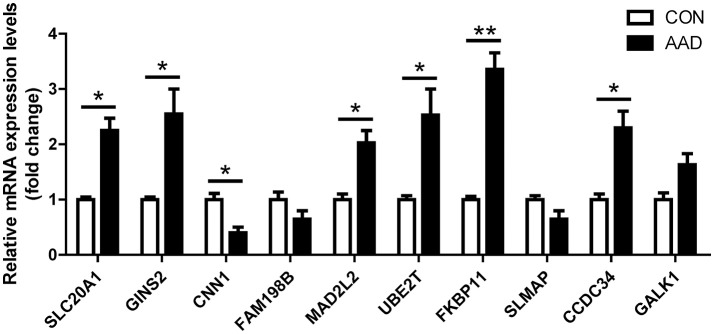
Validation of microarray data with qRT-PCR. The top 10 hub genes identified in microarray data validated by qRT-PCR is shown in bar graph. Total RNAs were isolated from aorta tissue of AAD patients and healthy donors, reverse-transcribed to cDNA and used as template for qRT-PCR analysis. Expression of each gene in aorta tissue was normalized with the respective healthy donors. CON, control healthy donor, open bars; AAD, patient with acute aortic dissection, black bars. Mean ± SEM of 6 subjects per group; ^*^P < 0.05, ^**^P < 0.01 (student *t*-test).

To obtain further evidence for the significance of the expression changes seen in the dodgerblue module, we evaluated the expression of the top 10 hub genes, *such as SLC20A1, GINS2, CNN1, FAM198B, MAD2L2, UBE2T, FKBP11, SLMAP, CCDC34*, and *GALK1 by* qRT-PCR in an independent sample set of 6 AAD cases and 6 controls originated from our study center (Table 1). Validating the hub genes module, we observed that many members of this module also showed significant differential expression changes between AAD and controls with the same directionality as in our discovery set. Overall, the qRT-PCR results were consistent with the results of the microarray analysis (Table 5). However, the qRT-PCR analysis tended to give higher up-regulation levels than those calculated from the microarray data. The highest change was found for *FKBP11* (Figure 4). To investigate the pathophysiological relevance of *FKBP11* gene as biomarker or interventional target for AAD, we performed additional *in vitro* and *ex vivo* analysis.

### FKBP11 expression increases in the aorta samples of the patients with aortic dissection

**Figure 5:**
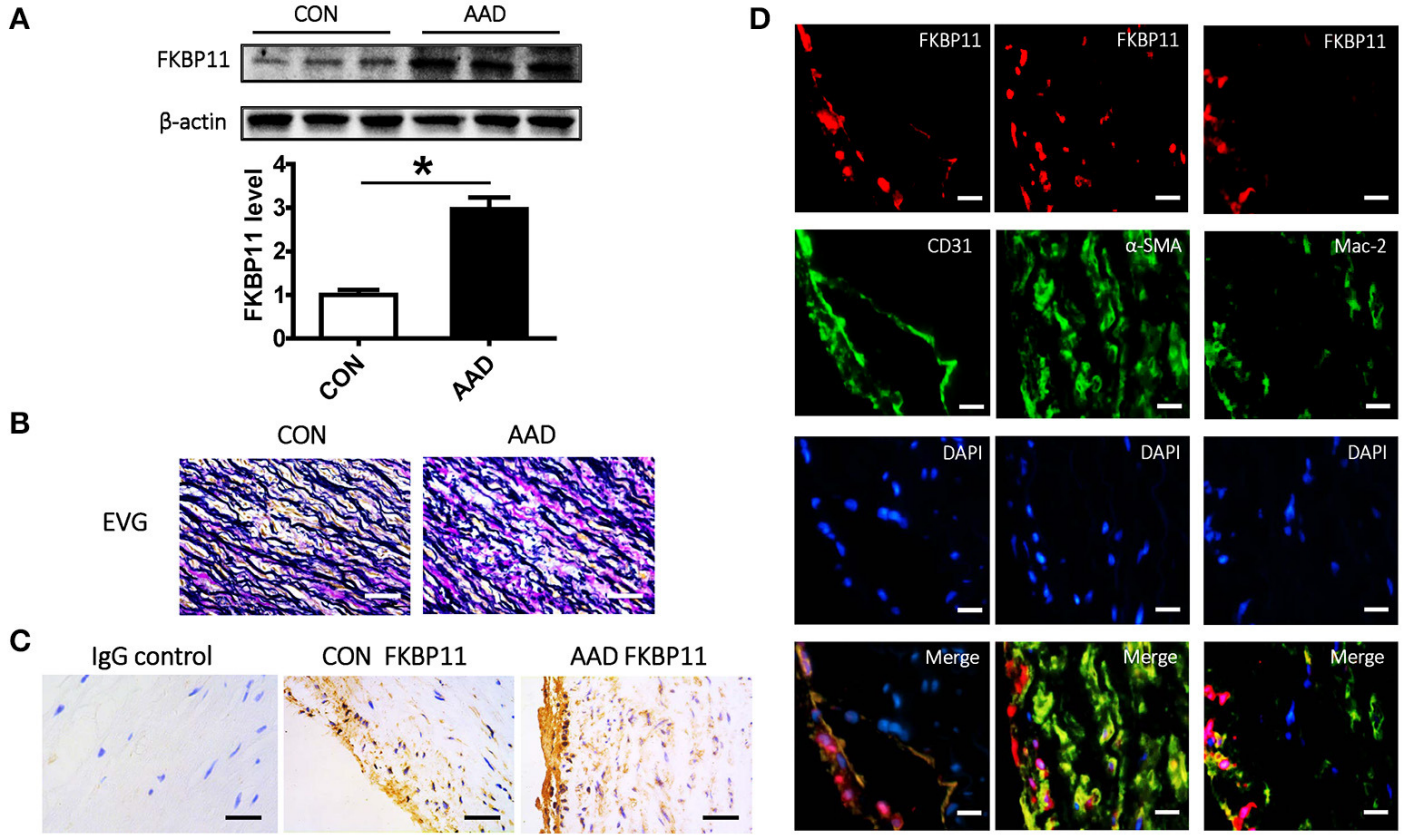
Expression and localization of FKBP11 in clinical aorta samples. **(A)** Representative immunoblots (upper panel) and bar graphs (lower panel) showing FKBP11 expression levels in whole aorta tissue lysates from AAD patients and control group. **(B)** Representative EVG staining of the aortic wall in the control group and AAD group. Distorted and deficient elastic fibers (dark blue staining) can be easily seen in AAD group, scale bar: 100 μm. **(C)** FKBP11 is strongly expressed in AAD group and in an endothelium prone manner. Exemplary immunohistochemical staining of aortic paraffin embedded tissue sections from control (left) and AAD (right) group; FKBP11 antigen detected by HRP-DAB reaction, brown; hematoxylin counterstain, blue; scale bar: 100 μm. **(D)** Double immunofluorescent staining showed co-localization of FKBP11 with endothelial marker CD31, not α-SMA (a smoot muscle cell marker) nor Mac-2 (a macrophage marker). And it showed that FKBP11 expression increased mainly on the endothelium of the AAD group. FKBP11 staining (red), nuclear staining DAPI (blue), specific cell marker (CD31, α-SMA and Mac-2) staining (green); scale bar: 100 μm. CON, control healthy donor, open bars; AADm patient with acute aortic dissection, black bars. Mean ± SEM of 6 subjects per group **(A-C)**; ^*^P < 0.05 (**A**, student *t*-test).

To ascertain the results of the above-described *in silico* analyses, we selected *FKBP11* as representative gene from the top 10 hub genes to determine its expression *ex vivo* in the aorta samples of the patients who underwent surgical procedure after aortic dissection. We determined the relative *FKBP11* gene expression with qRT-PCR and protein expression with Western Blot in the aorta samples. Consistent with the *in silico* analysis, the FKBP11 expression level was significantly higher at both mRNA and protein level as compared with to control samples (Figures 4, 5A). EVG staining of the aortic wall showed significantly distorted and deficient elastic fibers in AAD group (Figure 5B). Immunohistochemical staining of FKBP11 on the paraffin embedded aorta sections further proved the expression pattern and intriguingly revealed an endothelial prone staining of FKBP11 (Figure 5C). In order to determine the cell type specific expression of FKBP11, double immunofluorescent staining was performed. As we assumed, the expression of FKBP11 was mostly colocalized with an endothelial marker CD31 (Figure 5D). These data indicate that within aorta of AAD patients, the expression of FKBP11 is predominantly induced in the endothelium.

### FKBP11 correlates positively with the expression of MMP9 in infiltrated macrophages

**Figure 6:**
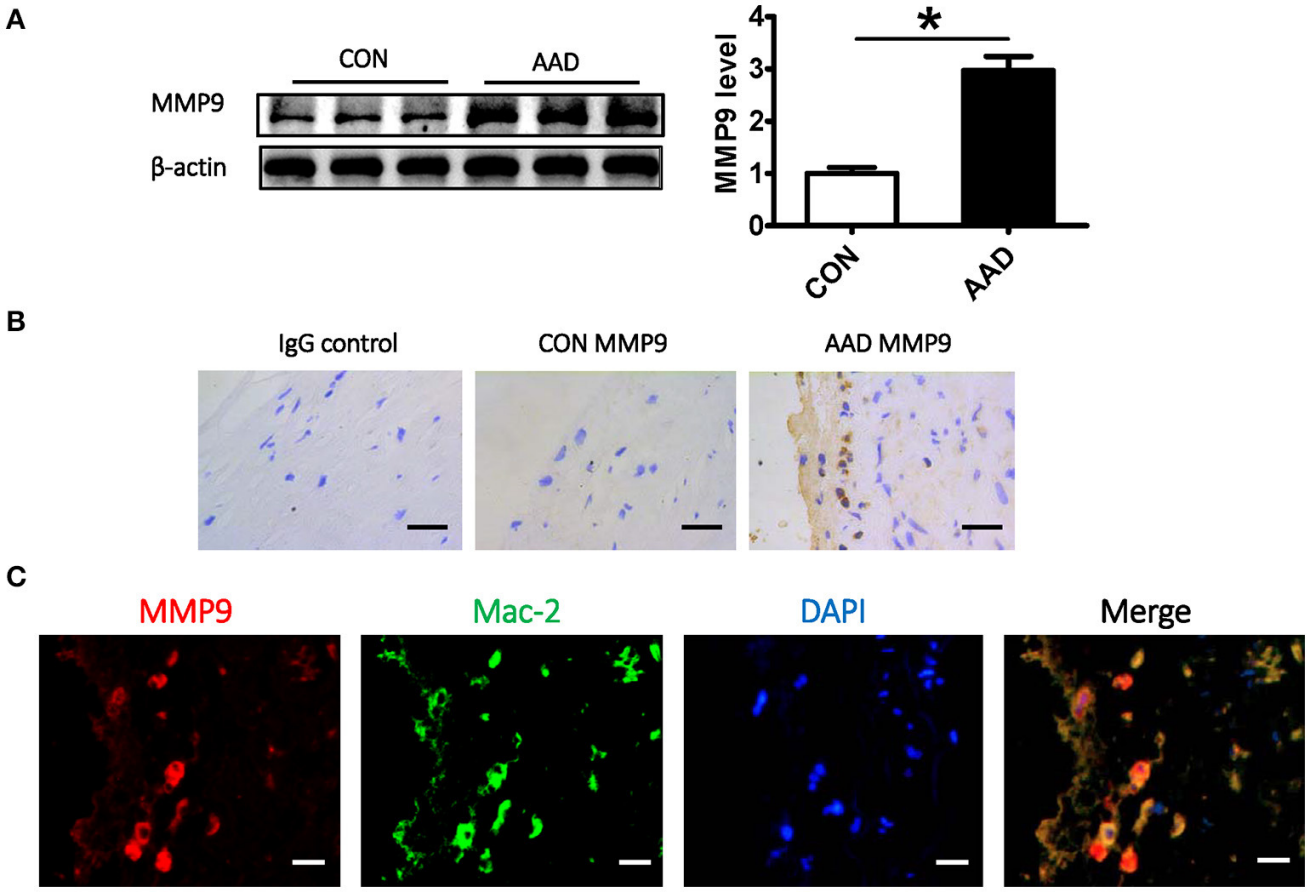
The correlation between FKBP11 and MMP9 expression in infiltrated macrophages under AAD. **(A)** Representative immune-blots (upper panel) and bar graphs (lower panel) showing MMP9 expression levels in whole aorta tissue lysates from AAD patients and control group. **(B)** Exemplary immune-histochemical staining of aortic paraffin embedded tissue sections from healthy donors and AAD patients; left, IgG control; middle, healthy donor; right, AAD patient; MMP9 antigen detected by HRP-DAB reaction, brown; hematoxylin counterstain, blue; scale bar: 100 μm. **(C)** Double immunofluorescent staining showed co-localization of MMP9 with macrophage marker Mac-2. It showed that the expression of MMP9 in infiltrated macrophages increased in AAD group. MMP9 staining (red), nuclear stain DAPI (blue), macrophage marker (Mac-2) staining (green); scale bar: 100 μm. CON, control healthy donor, open bars; AAD, patient with acute aortic dissection, black bars. Mean ± SEM of 6 subjects per group **(A–C)**; ^*^P < 0.05 (**A**, student *t*-test).

Matrix metalloproteinases (MMPs), especially MMP2 and MMP9, have been shown to be induced during the onset of aortic disease including AAD, promoting degradation of extracellular matrix components directly, such as elastin (Wu Z. et al., 2016). Western Blot analysis and immunohistochemical staining of MMP9 on clinical aorta samples showed that its expression was significant higher in AAD group (Figures 6A,B), which was consistent with the literature. In addition, MMP9 expression is positively correlated with the FKBP11 expression. But unlike the expression pattern of FKBP11 in endothelium, MMP9 localized dominantly in the middle layer, where mostly smooth muscle cells and infiltrated macrophages exist. In order to trace the source of MMP9, double immunofluorescent staining was performed, Immunofluorescence staining showed that MMP9 colocalized exclusively with Mac-2 (Figure 6C), a macrophage marker (Gough et al., 2006; Shahzad et al., 2011). The increased expression of MMP9, predominantly in macrophages has also been implicated in acute vascular crises such as atherosclerotic plaque rupture, abdominal aortic aneurysm, and aortic dissection (Wu Z. et al., 2016). But how the increased FKBP11 expression on endothelium could promote the expression of MMP9 in infiltrated macrophages in AD requires further elucidation.

### FKBP11 induces a pro-inflammatory state in endothelial cells through NF-kB pathway

**Figure 7:**
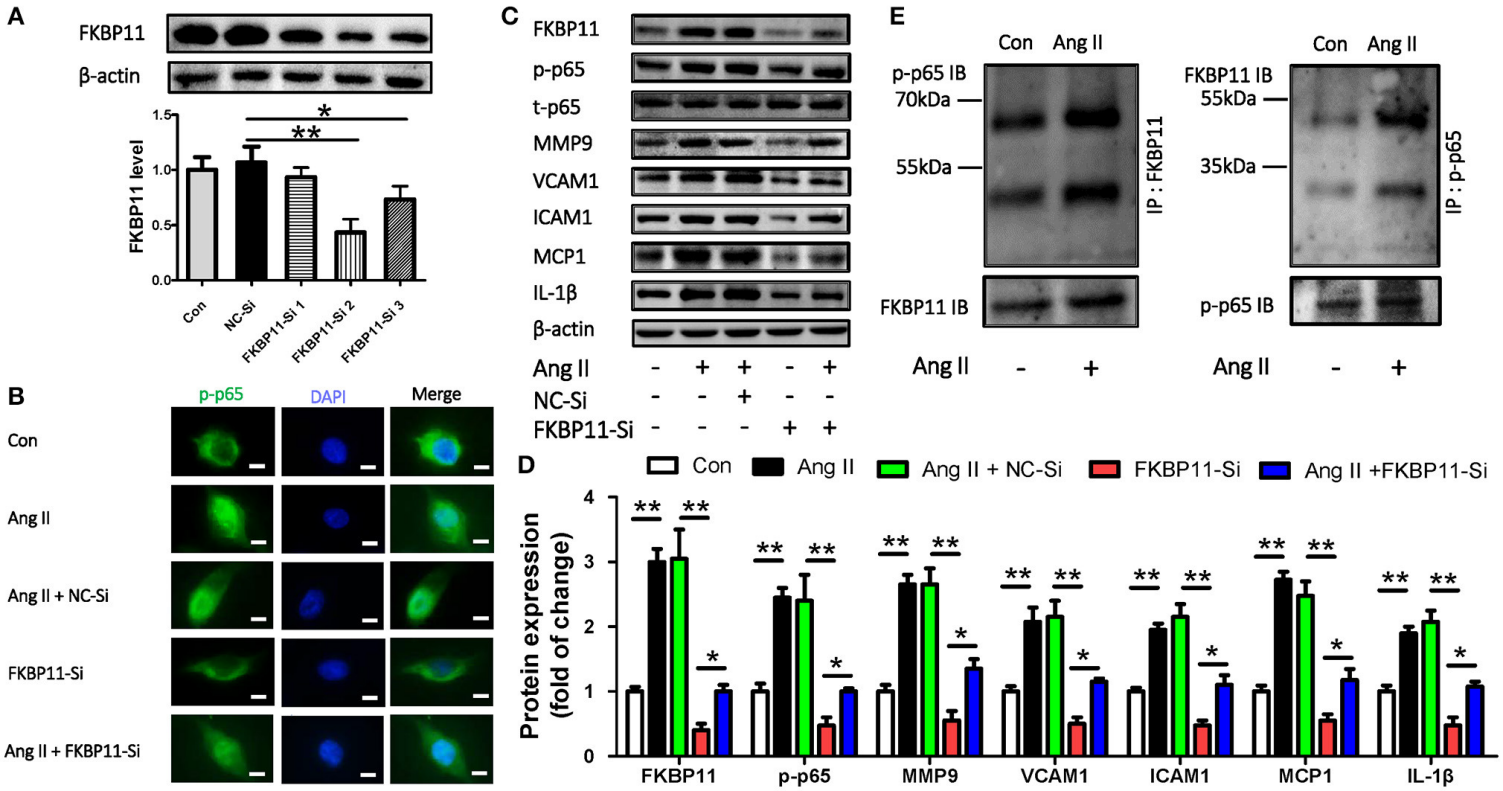
The pro-inflammatory function of FKBP11 and activation of NF-kB p65 subunit in endothelial cell. **(A)** Representative immunoblots (upper panel) and bar graph (lower panel) showed FKBP11 specific siRNA FKBP11-Si2 and FKBP11-Si3 but not FKBP11-Si1 can effectively suppress the protein expression as compared to the scrambled control NC-Si in EA.hy926 cells. **(B–D)** EA.hy926 cells were incubated with Ang II after transfected with FKBP11-Si2 or scrambled control NC-Si. **(B)** Cytofluorescent picture showed that Ang II promoted the nuclear localization of p-p65, while FKBP11 siRNA treatment could blunt it. Together, representative immunoblots **(C)** and bar graph **(D)** showing FKBP11-siRNA treatment could effectively suppress the phosphorylation and nuclear translocation of p65 and subsequently the expression of pro-inflammatory cytokines MCP1, VCAM1, ICAM1, and IL1-β. p-p65 staining (green); nuclear stain DAPI (blue); scale bar: 100 μm. **(E)** The interaction of FKBP11 and p-p65 was enhanced following Ang II treatment in EA.hy926 cells. Detection of p-p65 by immunoblotting following immunoprecipitation of FKBP11 from whole cell lysates of control (Con) cells or of cells after Ang II treatment (Ang II), left panel or vice versa, right panel; representative images of precipitated protein immunoblots (top) and respective input immunoblots (bottom). IgG controls for immunoprecipitation experiments were attached to the Supplementary Figure 3. CON: Control cells without treatment; Ang II: 1.0 × 10^−6^ mol/L Angiotensin II treated cells; NC-Si, Scrambled SiRNA for FKBP11; FKBP11-Si, FKBP11 knockdown SiRNA. Mean value ± SEM of at least three independent experiments **(A,D)**; ^*^P < 0.05; ^**^P < 0.01 (ANOVA); Representative immunoblots of at least three independent experiments; **(A,C,E)**; IB, immunoblot; IP, immunoprecipitation.

P65 subunit of NF-kB has been shown to provoke a pro-inflammatory state and induce MMPs and cytokines in aortic aneurysm and aortic dissection (Saito et al., 2013). In accordance with the literature, the expression of phospho-p65 was induced in a endothelial cell line EA.hy926 (ATCC® CRL-2922™) when treated with Angiotension II (Ang II), and the expression of MMP9 and other pro-inflammatory factors such as MCP1, VCAM1, ICAM1, and IL1beta were simultaneously augmented (Tieu et al., 2009). We hypothesize that the overexpression of FKBP11 could activate the endothelium through a p65 associated pathway. To further determine the role of FKBP11 in aortic dissection, we applied FKBP11 specific siRNA to knock down the expression in EA.hy926. As shown in Figure 7A, FKBP11-Si2 and FKBP11-Si3 can effectively suppress the protein expression as compared to the scrambled control. Importantly, FKBP11 knockdown suppressed the phosphorylation and nuclear translocation of p65 and subsequently the expression of the pro-inflammatory cytokines (Figures 7B–D). Intriguingly, by searching the Bio-GRID database (The Biological General Repository for Interaction Datasets: https://thebiogrid.org), which is an open access database dedicated to the annotation and archival of protein, genetic and chemical interactions for all major model organism species and humans, we found an association between FKBP11 and p65 (Chatr-Aryamontri et al., 2017). This association has been indicated by a yeast two-hybrid technology in a study mapping the interactions of human liver proteins (Wang et al., 2011). However, a functional study to validate this interaction is hitherto unknown. And here using co-immunoprecipitation assays, we demonstrate the direct interaction of these two proteins *in vitro*; in addition, this interaction is further augmented by Ang II treatment (Figure 7E). Importantly, Pan et al. (2014) found *JAK2* as key hub gene in AAD based on the same microarray data, however knockdown of FKBP11 in EA.hy926 resulted in unaltered expression of JAK2 regardless of Ang II treatment (Supplementary Figure 4). Furthermore, we could recapitulate the above findings in a primary endothelial cells HUVECs (Supplementary Figure 5). Hence, we could posit that in aortic dissection the increased expression of FKBP11 could elicit the pro-inflammatory state of the endothelium by interacting with NF-kB p65 subunit and promoting its nuclear translocation.

### FKBP11 knockdown suppresses the transmigration of the monocytes *in vitro*

**Figure 8:**
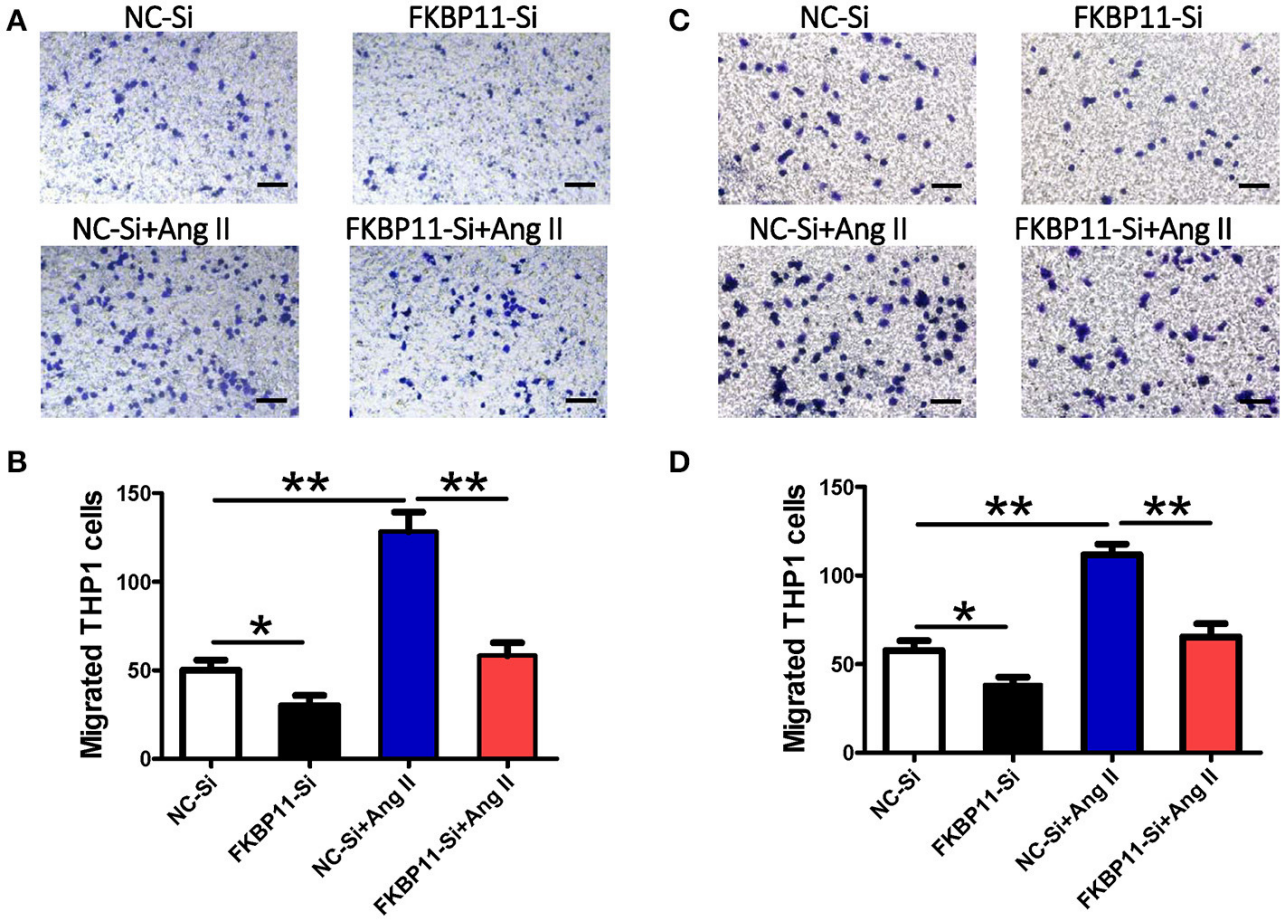
FKBP11 and monocyte transmigration through an endothelial cell monolayer. **(A,B)** EA.hy926 monolayer in the lower compartment was incubated without or with Ang II after transfected with FKBP11-Si RNA or NC–Si RNA for 24 h. Then 5 × 10^5^ THP-1 cells were seeded into each upper compartment containing 100 μL serum-free RPMI medium. Images depicting crystal violet stained THP-1 cells which migrated across the membrane of transwell inserts (pore size 8 μm) to the lower side after incubation with conditioned media from the lower compartment for 12 h. Representative pictures are shown. **(A)** Migrated cells per high power field were quantified and the data were showed in bar graph **(B)**. **(C,D)** The same as in **(A,B)**, instead of EA.hy926 cell monolayer, HUVECs were applied. NC-Si: Scrambled SiRNA for FKBP11 treated endothelial cells; FKBP11-Si: FKBP11 knockdown SiRNA treated endothelial cells; NC-Si+Ang II: Scrambled SiRNA for FKBP11 and 1.0 × 10^−6^ mol/L Angiotensin II treated endothelial cells; FKBP11-Si+AngII: FKBP11 knockdown SiRNA and 1.0 × 10^−6^ mol/L Angiotensin II treated endothelial cells. Mean value ± SEM of at least three independent experiments **(B,D)**; ^*^P < 0.05; ^**^P < 0.01 (ANOVA); Scale bar: 200 μm.

As we showed in Figure 7B, the increased FKBP11 expression on endothelium could promote the expression of MMP9 in infiltrated macrophages in AD. In addition, we found that increased expression of FKBP11 could elicit endothelial cell inflammation. Based on these data, we speculate that the inflamed endothelium could promote the transmigration of the circulating monocytes to the middle layer of the aorta and accelerate the formation and progression of aortic dissection. To ascertain this hypothesis, we performed a transwell experiment (Seehaus et al., 2009). Ang II treatment induced transmigration of THP1 cells, a monocytic cell line, more toward the endothelial monolayer when treated with Ang II. However, knockdown of the FKBP11 expression in endothelial cells abrogated the Ang II induced transmigration of THP1 cells (Figures 8A–D).

**Figure 9:**
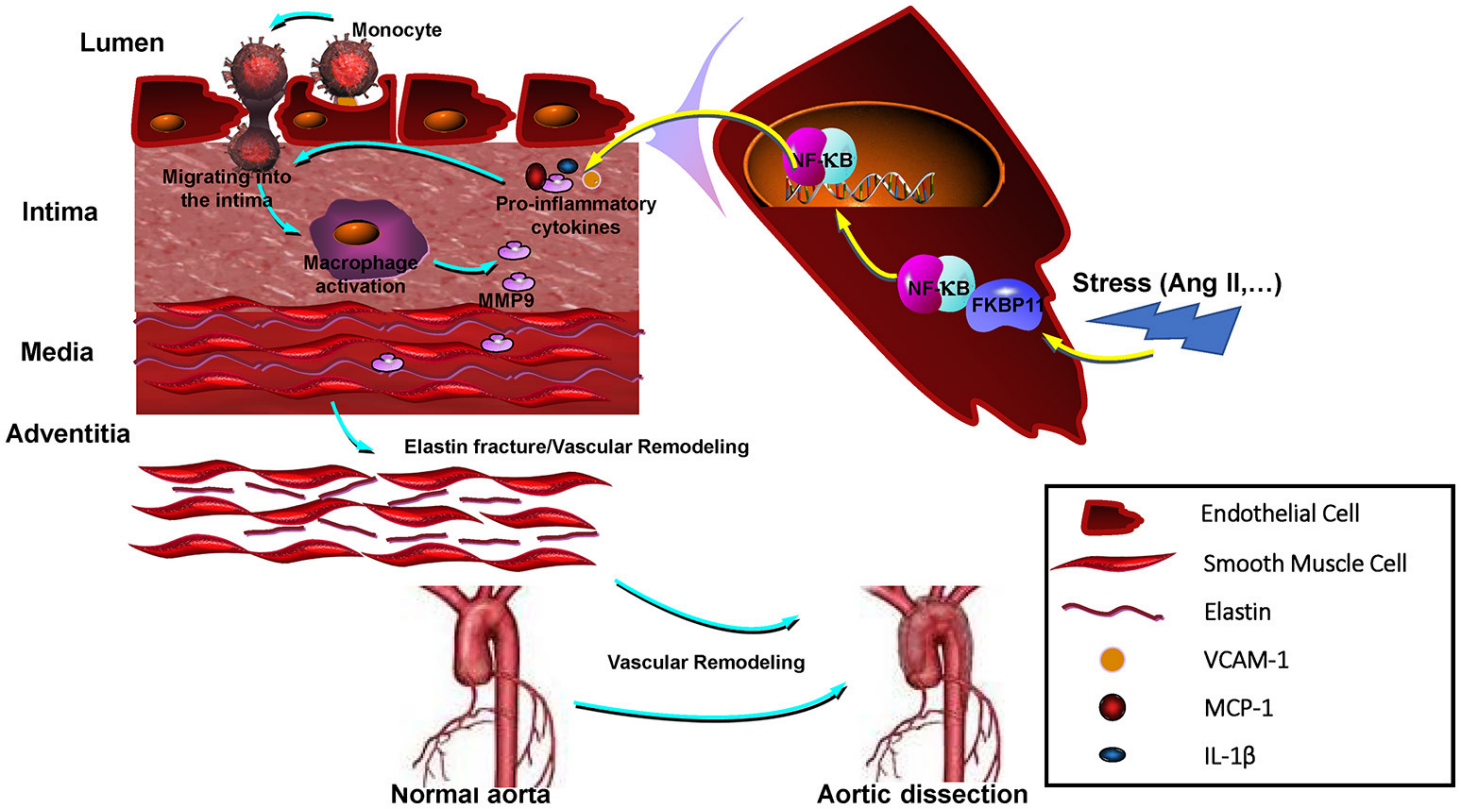
Proposed model of how FKBP11 affects the formation and progression of AAD. In response to environmental stress (such as Ang II), FKBP11 expression was induced in endothelium of the dissected aorta and it could provoke the pro-inflammatory state of the endothelial cells by interacting with NF-kB p65 subunit and promoting its nuclear translocation, and the produced pro-inflammatory cytokines further facilitated the circulating monocytes transmigrating through the endothelium to the middle layer of the aorta, where they differentiated into active macrophages and secreted MMPs and other ECM degrading proteins, finally promoted the formation and progression of aortic dissection.

Taken together that the current study involving both WGCNA and complementary gene expression analysis identify *FKBP11* is a key player in aortic dissection. FKBP11 expression predominantly induced within the endothelium of the dissected aorta could provoke endothelial cells inflammation. The pro-inflammatory effects of FKBP11 are mediated through its interaction with NF-kB p65 subunit, thereby promoting its nuclear translocation and pro-inflammatory cytokine production, further facilitating transendothelial migration of the circulating monocytes to the middle layer of the aorta. The infiltrated monocytes differentiate into active macrophages that can propagate sustained local inflammation by secreting MMPs and other ECM degrading proteins, thus contributing to the formation and progression of aortic dissection (Figure 9).

## Discussion

The present study applied a new strategy, based on WGCNA, to investigate the molecular mechanisms underlying AAD. For enrichment analysis of pathways, DEGs were mainly involved in pathways such as focal adhesion, cell cycle, ECM-receptor interaction, DNA replication, HIF-1 signaling pathway, p53 signaling pathway, and regulation of actin cytoskeleton. From the functional enrichment and GO analysis, we found that the major biological processes differentially regulated in AAD are DNA replication, mitotic nuclear division, extracellular matrix organization, protein binding, and actin binding. These are functional annotations associated with the inflammatory responses and ECM remodeling.

Moreover, we identified 9 distinct co-expression modules based on the DEGs. By functional enrichment analysis genes clustered in dodgerblue module have the strongest positive correlation with AAD, and further enrichment function analysis revealed that the inflammatory responses and ECM remodeling were involved in AAD progression and formation. Then a PPI (protein protein interaction) map was constructed based on the DEGs in the dodgerblue module. Top 10 hub genes including *SLC20A1, GINS2, CNN1, FAM198B, MAD2L2, UBE2T, FKBP11, SLMAP, CCDC34*, and *GALK1* were selected and qRT-PCR analysis was performed to verify their expression in an independent sample set. Our results demonstrate that the expression was consistent with the bioinformatics analysis results. Wherein the most obvious change of hub genes was *FKBP11*, whose expression was significantly up-regulated in AAD patients.

Subsequently, *in vitro* and *ex vivo* analyses were performed to further confirm the role of *FKBP11* in AAD. Our results illustrate that FKBP11 expression was induced in endothelium of the dissected aorta. In addition, it could provoke endothelial inflammation. This effect of FKBP11 is mediated through its interaction with NF-kB p65 subunit. The interaction thereby promotes nuclear translocation of the NF-kB p65 subunit and pro-inflammatory cytokines production. These cytokines further facilitate transendothelial migration of the circulating monocytes to the middle layer of the aorta. The infiltrated monocytes differentiate into active macrophages and secrete MMPs and other ECM degrading proteins. Subsequently, the secreted bioactive proteins contribute to the formation and progression of aortic dissection. Our study for the first time links FKBP11 with AAD and makes it a potential candidate for clinical diagnosis and treatment of AAD.

Although the microarray data which we adopted in the study was deposited by Pan et al. (2014), we further improve the analysis by the following aspects: (1) instead of integrative network analysis, Gene set enrichment analysis (GSEA) Hotspots/Expression Modules analysis, we used the much advanced WGCNA, which has been widely applied in microarray data analysis, such as in chronic obstructive pulmonary disease(COPD) (Obeidat et al., 2017), acute myocardial infarction(AMI) (Chen et al., 2016) and intracranial aneurysm(IA) (Zheng et al., 2015), by which we identified more pathways and hub genes involved in AAD; (2) the screened hub genes were verified with the same samples that were used for microarray experiment in the original study, of note we did in an independent sample set obtained from our institute; (3) we excluded an outlier in the AAD group based on our statistical analysis. However, in both studies we found inflammatory responses and related genes were tightly associated with AAD, which is in consistent with the literature, especially pro-inflammatory cytokines such as IL-6/IL-6R, CXCL1, and MCP1 were involved in vascular inflammation and aortic dissection (Tieu et al., 2009; Ju et al., 2013; Anzai et al., 2015). Besides, some screened hub genes were common, like *CNN1*, an actin filament-associated regulatory protein expressed in smooth muscle and many types of non-muscle cells(Liu and Jin, 2016), and *CCDC 34*, involved in bladder carcinoma pathogenesis (Gong et al., 2015) and non-small-cell lung cancer (Petroziello et al., 2004). Nevertheless, we found some other different hub genes, e.g., *SLC20A1*, involved in vascular smooth muscle cell calcification (Wu et al., 2016) and calcific aortic valve disease (El Husseini et al., 2013); *MAD2L2*, a component of the mitotic spindle assembly checkpoint and a crucial contributor to the control of DNA repair activity (Boersma et al., 2015); *UBE2T*, a ubiquitin-conjugating enzyme (E2) essential for Fanconi anemia and prostate cancer (Machida et al., 2006; Wen et al., 2015); *SLMAP*, associated with Brugada syndrome and diabetes (Ishikawa et al., 2012; Upadhyay et al., 2015); *GALK1*, a major enzyme for the metabolism of galactose (Sangiuolo et al., 2004);*GINS2*, a complex essential in the initiation of DNA replication and progression of DNA replication fork (Liang et al., 2016). Additionally, role for *FAM198B* in AAD is hitherto unknown.

Remarkably, the key hub gene in AAD found in the original study was *JAK2*, which plays a key role in the inflammatory process. In line with the pro-inflammatory role of *JAK2*, we discovered *FKBP11*, independent of *JAK2*, as key player in AAD in our current study. *FKBP11* belongs to the FKBP family of peptidyl-prolyl cis/trans isomerases, which catalyze the folding of proline-containing polypeptides and whose activity could be inhibited by the immunosuppressant compounds FK506 and rapamycin (Rulten et al., 2006). The distinct results may come from the improved analytical methodology and the optimized microarray data.

Taken together, these results indicate that *FKBP11* could be a key regulator in vascular inflammation in AAD. Indeed, in accordance with our current data, *FKBP11* has already been shown to be involved in inflammatory regulation, such as B cell tolerance and differentiation (Ruer-Laventie et al., 2015) and osteoblast interferon-inducible genes induction (Hanagata and Li, 2011). Thus, it was of particular interest to further discuss its potential biological function. Here we could show that the expression of FKBP11 (mRNA and Protein) was induced in AAD aortic samples, especially in vascular endothelium. As shown in literature, other FKBP family members like FKBP51 and FKBP 52 could directly bind p65 subunit and be recruited to the promoter sites of NF-kB target genes and affect the expression of pro-inflammatory cytokines (Erlejman et al., 2014). Along this line and considering the structural similarities of these FKBP proteins (Rulten et al., 2006), we hypothesized that FKBP11 could also bind NF-kB p65 subunit and regulate the expression of pro-inflammatory cytokine in endothelial cells. Accordingly, co-immunoprecipitation assay showed that FKBP11 did interact with p65, and its overexpression was associated with the phosphorylation of p65 and subsequently induction of pro-inflammatory cytokines, such as MMP9, MCP1, ICAM1, VCAM1, and IL1beta, and this effect could be inhibited by siRNA knockdown *in vitro*. However, currently it is not known whether FKBP11 directly interacts with p65 subunit or requires involvement of other adapter proteins. Based on the structural homology with other FKBP proteins it is quite possible that FKBP11 and p65, may form a complex together and then be recruited to the specific promoter regions of the cytokine genes. However, this remains a subject of future investigations. Within the current study, we found that upregulation of FKBP11 was associated with the enhanced macrophage MMP9 expression in the midlayer of the AAD samples, which could be an explanation for the formation and progression of AAD. And this hypothesis was further verified with an *in vitro* transmigration assay, in which we showed that Angiotensin II could promote the transmigration of the monocytic cell THP1 toward the endothelial cell layer, while knockdown of FKBP11 in the endothelial cells significantly blunted the effect of Angiotensin II.

We speculate that the pro-inflammatory cytokines, such as MCP1, ICAM1, VCAM1, IL1beta, and etc., secreted by the endothelial cells could act as chemokines and promote the circulating monocytes to transmigrate through the endothelial layer and differentiate into active macrophages in the midlayer, where they produced various factors like MMP9, which compromises the integrity of the vascular wall resulting in AAD. This hypothesis is further supported by several other studies (Tham et al., 2002; Caird et al., 2006; Tieu et al., 2009; Wu et al., 2010). Recently another member of this family FKBP10 has been shown to play a role in collagen processing and might thus modulate ECM composition in interstitial fibroblasts in idiopathic pulmonary fibrosis (IPF) (Staab-Weijnitz et al., 2015). Based on these studied, it can be assumed that FKBP11 also might regulate collagen expression and thereafter ECM stability in the interstitial fibroblasts in AAD; however, the endothelium specific expression pattern of FKBP11 could exclude this possibility.

In summary, through weighted gene co-expression network analysis (WGCNA), we uncovered *FKBP11* as a key hub gene in AAD. Our data suggest that FKBP11 can be potential target for AAD.

## Study's limitations

It is important to emphasize the main limitations of our study. First, the original sample size is a bit low (6 AAD + 5 Control, normally >6), and the sex (most males), age and some other important parameters were not normalized. However, we verified the top 10 hub genes with an independent sample set from our institution. Second, we do not have access to the first hand raw data; the related circumstances which could affect the assay were unknown. This analysis is based on whole aorta tissues gene expression profile, yet, tissue-specific or even single cell transcriptome analyses could give us much more accurate understanding of the pathophysiology of AAD. Third, we mainly focus on the role of FKBP11 in our study, considering the similar structure and function of the other FKBP family members, like FKBP10, FKBP51, and FKBP52, their roles were not excluded and warrants additional investigation. Fourth, the functional study was executed with only *in vitro* and *ex vivo* analysis, further *in vivo* experiment, e.g., endothelial cell specific knock out of FKBP11 mouse aortic dissection model is required to strengthen our points.

## Author contributions

TW together with HW designed and conducted *in vitro* work, clinical sample collection, *ex vivo* analysis; WZ, RT, LX, and XL assisted in clinical sample collection and *ex vivo* analysis. XH, WL, YL, and QH provided reagents, conceptual advice, and critically reviewed the manuscript; HW and HZ conceptually designed the study and prepared the manuscript.

### Conflict of interest statement

The authors declare that the research was conducted in the absence of any commercial or financial relationships that could be construed as a potential conflict of interest.

## References

[B1] Anzai A. Shimoda M. Endo J. Kohno T. Katsumata Y. Matsuhashi T.. (2015). Adventitial CXCL1/G-CSF expression in response to acute aortic dissection triggers local neutrophil recruitment and activation leading to aortic rupture. Circ. Res. 116, 612–623. 10.1161/CIRCRESAHA.116.304918 25563839

[B2] Boersma V. Moatti N. Segura-Bayona S. Peuscher M. H. van der Torre J. Wevers B. A.. (2015). MAD2L2 controls DNA repair at telomeres and DNA breaks by inhibiting 5' end resection. Nature 521, 537–540. 10.1038/nature14216 25799990 PMC4481296

[B3] Bolstad B. M. Irizarry R. A. Astrand M. Speed T. P. (2003). A comparison of normalization methods for high density oligonucleotide array data based on variance and bias. Bioinformatics 19, 185–193. 10.1093/bioinformatics/19.2.185 12538238

[B4] Caird J. Napoli C. Taggart C. Farrell M. Bouchier-Hayes D. (2006). Matrix metalloproteinases 2 and 9 in human atherosclerotic and non-atherosclerotic cerebral aneurysms. Eur. J. Neurol. 13, 1098–1105. 10.1111/j.1468-1331.2006.01469.x 16987162

[B5] Chatr-Aryamontri A. Oughtred R. Boucher L. Rust J. Chang C. Kolas N. K.. (2017). The BioGRID interaction database: 2017 update. Nucleic Acids Res. 45, D369–D379. 10.1093/nar/gkw1102 27980099 PMC5210573

[B6] Chen J. Yu L. Zhang S. Chen X. (2016). Network analysis-based approach for exploring the potential diagnostic biomarkers of acute myocardial infarction. Front. Physiol. 7:615. 10.3389/fphys.2016.00615 28018242 PMC5145872

[B7] Dong W. Wang H. Shahzad K. Bock F. Al-Dabet M. M. Ranjan S.. (2015). Activated protein C ameliorates renal ischemia-reperfusion injury by restricting Y-box binding protein-1 ubiquitination. J. Am. Soc. Nephrol. 26, 2789–2799. 10.1681/ASN.2014080846 26015455 PMC4625674

[B8] El Husseini D. Boulanger M. C. Fournier D. Mahmut A. Bosse Y. Pibarot P.. (2013). High expression of the Pi-transporter SLC20A1/Pit1 in calcific aortic valve disease promotes mineralization through regulation of Akt-1. PLoS ONE 8:e53393. 10.1371/journal.pone.0053393 23308213 PMC3537628

[B9] Erlejman A. G. De Leo S. A. Mazaira G. I. Molinari A. M. Camisay M. F. Fontana V.. (2014). NF-κB transcriptional activity is modulated by FK506-binding proteins FKBP51 and FKBP52: a role for peptidyl-prolyl isomerase activity. J. Biol. Chem. 289, 26263–26276. 10.1074/jbc.M114.582882 25104352 PMC4176250

[B10] Goh K. I. Cusick M. E. Valle D. Childs B. Vidal M. Barabasi A. L. (2007). The human disease network. Proc. Natl. Acad. Sci. U.S.A. 104, 8685–8690. 10.1073/pnas.0701361104 17502601 PMC1885563

[B11] Gong Y. Qiu W. Ning X. Yang X. Liu L. Wang Z.. (2015). CCDC34 is up-regulated in bladder cancer and regulates bladder cancer cell proliferation, apoptosis and migration. Oncotarget 6, 25856–25867. 10.18632/oncotarget.4624 26312564 PMC4694871

[B12] Gough P. J. Gomez I. G. Wille P. T. Raines E. W. (2006). Macrophage expression of active MMP-9 induces acute plaque disruption in apoE-deficient mice. J. Clin. Invest. 116, 59–69. 10.1172/JCI25074 16374516 PMC1319218

[B13] Hanagata N. Li X. (2011). Osteoblast-enriched membrane protein IFITM5 regulates the association of CD9 with an FKBP11-CD81-FPRP complex and stimulates expression of interferon-induced genes. Biochem. Biophys. Res. Commun. 409, 378–384. 10.1016/j.bbrc.2011.04.136 21600883

[B14] Huang da W. Sherman B. T. Lempicki R. A. (2009a). Bioinformatics enrichment tools: paths toward the comprehensive functional analysis of large gene lists. Nucleic Acids Res. 37, 1–13. 10.1093/nar/gkn923 19033363 PMC2615629

[B15] Huang da W. Sherman B. T. Lempicki R. A. (2009b). Systematic and integrative analysis of large gene lists using DAVID bioinformatics resources. Nat. Protoc. 4, 44–57. 10.1038/nprot.2008.211 19131956

[B16] Ishikawa T. Sato A. Marcou C. A. Tester D. J. Ackerman M. J. Crotti L.. (2012). A novel disease gene for Brugada syndrome: sarcolemmal membrane-associated protein gene mutations impair intracellular trafficking of hNav1.5. Circ. Arrhythm. Electrophysiol. 5, 1098–1107. 10.1161/CIRCEP.111.969972 23064965

[B17] Ju X. Ijaz T. Sun H. Ray S. Lejeune W. Lee C.. (2013). Interleukin-6-signal transducer and activator of transcription-3 signaling mediates aortic dissections induced by angiotensin II via the T-helper lymphocyte 17-interleukin 17 axis in C57BL/6 mice. Arterioscler. Thromb. Vasc. Biol. 33, 1612–1621. 10.1161/ATVBAHA.112.301049 23685554 PMC3818154

[B18] Langfelder P. Horvath S. (2008). WGCNA: an R package for weighted correlation network analysis. BMC Bioinformatics 9:559. 10.1186/1471-2105-9-559 19114008 PMC2631488

[B19] Liang P. Song Z. Chen D. Linghu R. Wang Y. Zhang X. (2016). GINS2 regulates matrix metallopeptidase 9 expression and cancer stem cell property in human triple negative Breast cancer. Biomed. Pharmacother. 84, 1568–1574. 10.1016/j.biopha.2016.10.032 27829549

[B20] Liu J. Li Y. Liu L. Wang Z. Shi C. Cheng Z.. (2017). Double Knockdown of PHD1 and Keap1 Attenuated Hypoxia-Induced Injuries in Hepatocytes. Front. Physiol. 8:291. 10.3389/fphys.2017.00291 28539891 PMC5423937

[B21] Liu R. Jin J. P. (2016). Calponin isoforms CNN1, CNN2 and CNN3: regulators for actin cytoskeleton functions in smooth muscle and non-muscle cells. Gene 585, 143–153. 10.1016/j.gene.2016.02.040 26970176 PMC5325697

[B22] Liu W. Huang X. Liang X. Zhou Y. Li H. Yu Q.. (2017). Identification of key modules and hub genes of keloids with weighted gene coexpression network analysis. Plast. Reconstr. Surg. 139, 376–390. 10.1097/PRS.0000000000003014 28121871

[B23] Machida Y. J. Machida Y. Chen Y. Gurtan A. M. Kupfer G. M. D'Andrea A. D.. (2006). UBE2T is the E2 in the Fanconi anemia pathway and undergoes negative autoregulation. Mol. Cell 23, 589–596. 10.1016/j.molcel.2006.06.024 16916645

[B24] Madhusudhan T. Wang H. Dong W. Ghosh S. Bock F. Thangapandi V. R.. (2015). Defective podocyte insulin signalling through p85-XBP1 promotes ATF6-dependent maladaptive ER-stress response in diabetic nephropathy. Nat. Commun. 6:6496. 10.1038/ncomms7496 25754093 PMC4366504

[B25] Madhusudhan T. Wang H. Straub B. K. Grone E. Zhou Q. Shahzad K.. (2012). Cytoprotective signaling by activated protein C requires protease-activated receptor-3 in podocytes. Blood 119, 874–883. 10.1182/blood-2011-07-365973 22117049 PMC3398751

[B26] Meszaros I. Morocz J. Szlavi J. Schmidt J. Tornoci L. Nagy L.. (2000). Epidemiology and clinicopathology of aortic dissection. Chest 117, 1271–1278. 10.1378/chest.117.5.1271 10807810

[B27] Obeidat M. Nie Y. Chen V. Shannon C. P. Andiappan A. K. Lee B.. (2017). Network-based analysis reveals novel gene signatures in peripheral blood of patients with chronic obstructive pulmonary disease. Respir. Res. 18:72. 10.1186/s12931-017-0558-1 28438154 PMC5404332

[B28] Ohtani K. Egashira K. Hiasa K. Zhao Q. Kitamoto S. Ishibashi M.. (2004). Blockade of vascular endothelial growth factor suppresses experimental restenosis after intraluminal injury by inhibiting recruitment of monocyte lineage cells. Circulation 110, 2444–2452. 10.1161/01.CIR.0000145123.85083.66 15477409

[B29] Olsson C. Thelin S. Stahle E. Ekbom A. Granath F. (2006). Thoracic aortic aneurysm and dissection: increasing prevalence and improved outcomes reported in a nationwide population-based study of more than 14,000 cases from 1987 to 2002. Circulation 114, 2611–2618. 10.1161/CIRCULATIONAHA.106.630400 17145990

[B30] Pan S. Wu D. Teschendorff A. E. Hong T. Wang L. Qian M.. (2014). JAK2-centered interactome hotspot identified by an integrative network algorithm in acute Stanford type A aortic dissection. PLoS ONE 9:e89406. 10.1371/journal.pone.0089406 24586754 PMC3933461

[B31] Petroziello J. Yamane A. Westendorf L. Thompson M. McDonagh C. Cerveny C.. (2004). Suppression subtractive hybridization and expression profiling identifies a unique set of genes overexpressed in non-small-cell lung cancer. Oncogene 23, 7734–7745. 10.1038/sj.onc.1207921 15334068

[B32] Ruer-Laventie J. Simoni L. Schickel J. N. Soley A. Duval M. Knapp A. M.. (2015). Overexpression of Fkbp11, a feature of lupus B cells, leads to B cell tolerance breakdown and initiates plasma cell differentiation. Immun. Inflamm. Dis. 3, 265–279. 10.1002/iid3.65 26417441 PMC4578525

[B33] Rulten S. L. Kinloch R. A. Tateossian H. Robinson C. Gettins L. Kay J. E. (2006). The human FK506-binding proteins: characterization of human FKBP19. Mamm. Genome 17, 322–331. 10.1007/s00335-005-0127-7 16596453

[B34] Saito T. Hasegawa Y. Ishigaki Y. Yamada T. Gao J. Imai J.. (2013). Importance of endothelial NF-kappaB signalling in vascular remodelling and aortic aneurysm formation. Cardiovasc. Res. 97, 106–114. 10.1093/cvr/cvs298 23015640

[B35] Sangiuolo F. Magnani M. Stambolian D. Novelli G. (2004). Biochemical characterization of two GALK1 mutations in patients with galactokinase deficiency. Hum. Mutat. 23:396. 10.1002/humu.9223 15024738

[B36] Seehaus S. Shahzad K. Kashif M. Vinnikov I. A. Schiller M. Wang H.. (2009). Hypercoagulability inhibits monocyte transendothelial migration through protease-activated receptor-1-, phospholipase-Cbeta-, phosphoinositide 3-kinase-, and nitric oxide-dependent signaling in monocytes and promotes plaque stability. Circulation 120, 774–784. 10.1161/CIRCULATIONAHA.109.849539 19687358

[B37] Shahzad K. Bock F. Al-Dabet M. M. Gadi I. Nazir S. Wang H.. (2016). Stabilization of endogenous Nrf2 by minocycline protects against Nlrp3-inflammasome induced diabetic nephropathy. Sci. Rep. 6:34228. 10.1038/srep34228 27721446 PMC5056367

[B38] Shahzad K. Thati M. Wang H. Kashif M. Wolter J. Ranjan S.. (2011). Minocycline reduces plaque size in diet induced atherosclerosis via p27(Kip1). Atherosclerosis 219, 74–83. 10.1016/j.atherosclerosis.2011.05.041 21719015

[B39] Smyth G. K. (2004). Linear models and empirical bayes methods for assessing differential expression in microarray experiments. Stat. Appl. Genet. Mol. Biol. 3:Article3. 10.2202/1544-6115.1027 16646809

[B40] Staab-Weijnitz C. A. Fernandez I. E. Knuppel L. Maul J. Heinzelmann K. Juan-Guardela B. M.. (2015). FK506-binding protein 10, a potential novel drug target for idiopathic pulmonary fibrosis. Am. J. Respir. Crit. Care Med. 192, 455–467. 10.1164/rccm.201412-2233OC 26039104 PMC4595665

[B41] Steuer J. Bjorck M. Mayer D. Wanhainen A. Pfammatter T. Lachat M. (2013). Distinction between acute and chronic type B aortic dissection: is there a sub-acute phase? Eur. J. Vasc. Endovasc. Surg. 45, 627–631. 10.1016/j.ejvs.2013.03.013 23602854

[B42] Szklarczyk D. Morris J. H. Cook H. Kuhn M. Wyder S. Simonovic M.. (2017). The STRING database in 2017: quality-controlled protein-protein association networks, made broadly accessible. Nucleic. Acids. Res. 45, D362–D368. 10.1093/nar/gkw937 27924014 PMC5210637

[B43] Tham D. M. Martin-McNulty B. Wang Y. X. Wilson D. W. Vergona R. Sullivan M. E.. (2002). Angiotensin II is associated with activation of NF-kappaB-mediated genes and downregulation of PPARs. Physiol. Genomics 11, 21–30. 10.1152/physiolgenomics.00062.2002 12361987

[B44] Tieu B. C. Lee C. Sun H. Lejeune W. Recinos A. III. Ju X.. (2009). An adventitial IL-6/MCP1 amplification loop accelerates macrophage-mediated vascular inflammation leading to aortic dissection in mice. J. Clin. Invest. 119, 3637–3651. 10.1172/JCI38308 19920349 PMC2786788

[B45] Upadhyay R. Robay A. Fakhro K. Abi Khalil C. Zirie M. Jayyousi A.. (2015). Role of SLMAP genetic variants in susceptibility of diabetes and diabetic retinopathy in Qatari population. J. Transl. Med. 13:61. 10.1186/s12967-015-0411-6 25880194 PMC4335364

[B46] Wang J. Huo K. Ma L. Tang L. Li D. Huang X.. (2011). Toward an understanding of the protein interaction network of the human liver. Mol. Syst. Biol. 7:536. 10.1038/msb.2011.67 21988832 PMC3261708

[B47] Wen M. Kwon Y. Wang Y. Mao J. H. Wei G. (2015). Elevated expression of UBE2T exhibits oncogenic properties in human prostate cancer. Oncotarget 6, 25226–25239. 10.18632/oncotarget.4712 26308072 PMC4694827

[B48] Wu G. Chen T. Shahsafaei A. Hu W. Bronson R. T. Shi G. P.. (2010). Complement regulator CD59 protects against angiotensin II-induced abdominal aortic aneurysms in mice. Circulation 121, 1338–1346. 10.1161/CIRCULATIONAHA.108.844589 20212283 PMC3057574

[B49] Wu Y. Han X. Wang L. Diao Z. Liu W. (2016). Indoxyl sulfate promotes vascular smooth muscle cell calcification via the JNK/Pit-1 pathway. Ren. Fail. 38, 1702–1710. 10.3109/0886022X.2016.1155397 27001263

[B50] Wu Z. Ruan Y. Chang J. Li B. Ren W. (2016). Angiotensin II is related to the acute aortic dissection complicated with lung injury through mediating the release of MMP9 from macrophages. Am. J. Transl. Res. 8, 1426–1436 27186269 PMC4859628

[B51] Yip A. M. Horvath S. (2007). Gene network interconnectedness and the generalized topological overlap measure. BMC Bioinformatics 8:22. 10.1186/1471-2105-8-22 17250769 PMC1797055

[B52] Zhang B. Horvath S. (2005). A general framework for weighted gene co-expression network analysis. Stat. Appl. Genet. Mol. Biol. 4:Article17. 10.2202/1544-6115.1128 16646834

[B53] Zhang C. van der Voort D. Shi H. Zhang R. Qing Y. Hiraoka S.. (2016). Matricellular protein CCN3 mitigates abdominal aortic aneurysm. J. Clin. Invest. 126, 1282–1299. 10.1172/JCI82337 26974158 PMC4811126

[B54] Zhao W. Langfelder P. Fuller T. Dong J. Li A. Hovarth S. (2010). Weighted gene coexpression network analysis: state of the art. J. Biopharm. Stat. 20, 281–300. 10.1080/10543400903572753 20309759

[B55] Zheng X. Xue C. Luo G. Hu Y. Luo W. Sun X. (2015). Identification of crucial genes in intracranial aneurysm based on weighted gene coexpression network analysis. Cancer Gene Ther. 22, 238–245. 10.1038/cgt.2015.10 25721208

