# Supplementary material for: Weighted Gene Co-expression Network Analysis Identifies FKBP11 as a Key Regulator in Acute Aortic Dissection through a NF-kB Dependent Pathway
Source: Front Physiol. 2017 Dec 4;8:1010. doi: 10.3389/fphys.2017.01010 (PMC5723018; doi:10.3389/fphys.2017.01010)
Supplement: Supplementary file 1 [file DataSheet1.doc]

Supplementary Material

# Weighted Gene Co-expression Network Analysis identifies FKBP11 as a key regulator in acute aortic dissection through a NF-kB dependent pathway

Tao Wang^1^, Xingwei He^1^, Xintian Liu^2^, Yujian Liu^1^, Wenjun Zhang^1^, Qiang Huang^1^, Wanjun Liu^1^, Luyang Xiong^1^, Rong Tan^3^, Hongjie Wang^1*^ and Hesong Zeng^1*^

*** Correspondence:**Hongjie Wang and Hesong Zeng [hongjie.wang@tjh.tjmu.edu.cn](mailto:hongjie.wang@tjh.tjmu.edu.cn) and [zenghs@tjh.tjmu.edu.cn](mailto:zenghs@tjh.tjmu.edu.cn)

**Supplementary Figure 1**

**
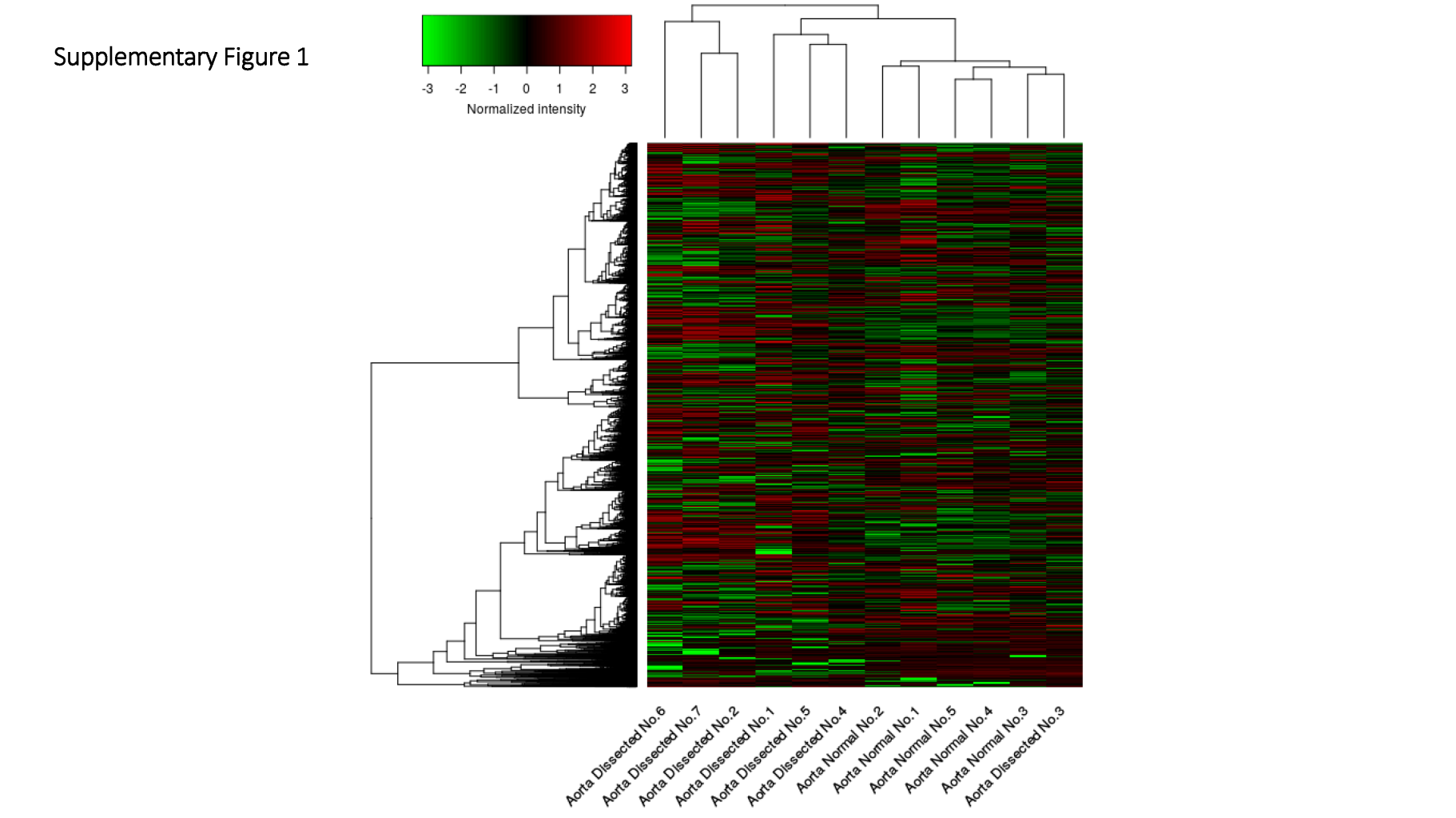
**

**Supplementary Figure 2.** DEGs can be effectively divided into AAD and control groups except Aorta Dissected NO. 3. Red indicates that the gene that is upregulated and green represents down-regulated genes.

**Supplementary Figure 2**


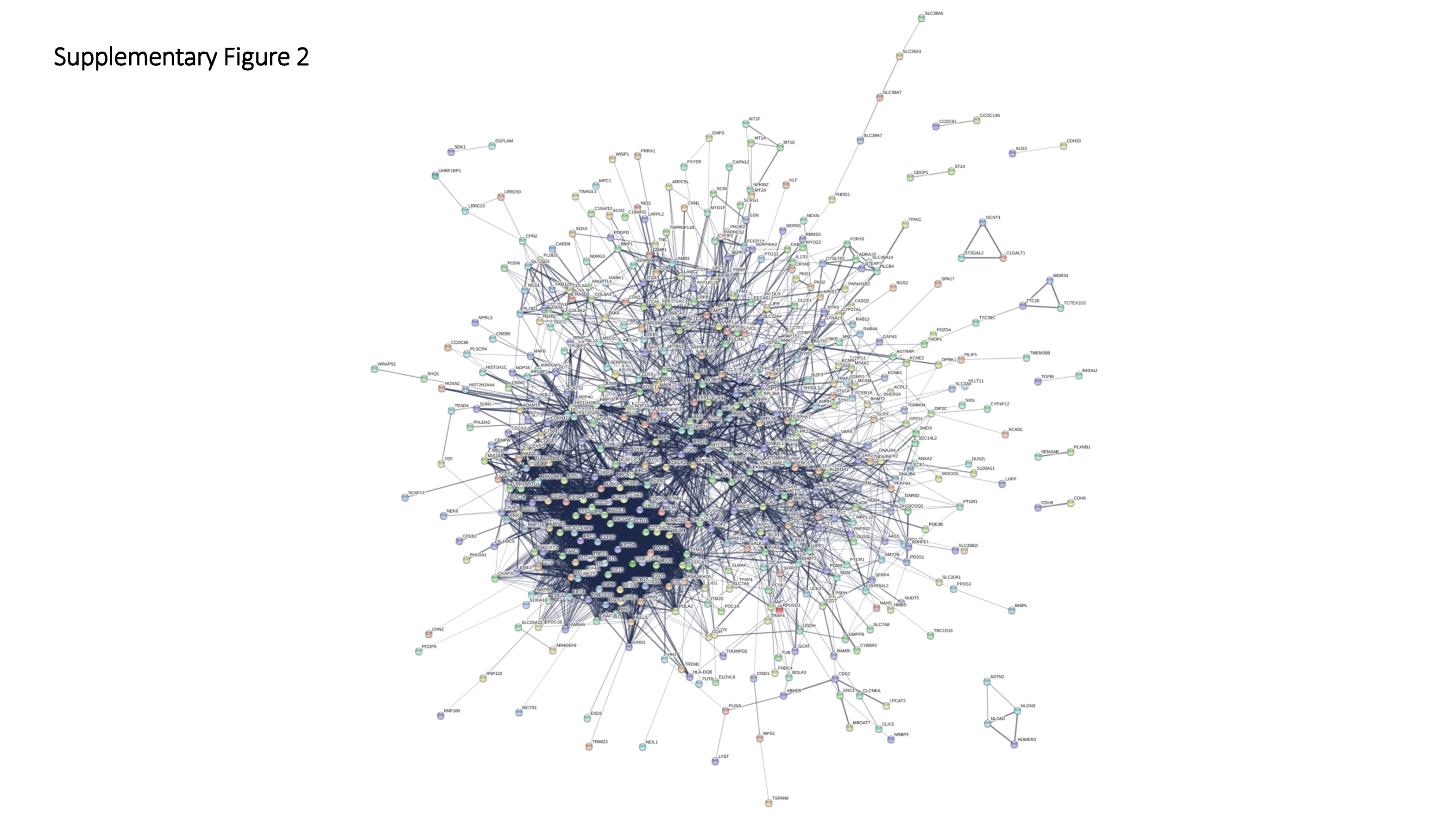
 **Supplementary Figure 2.** PPI (protein protein interaction) network was constructed based on the DEGs in the dodgerblue module.

**Supplementary Figure 3**


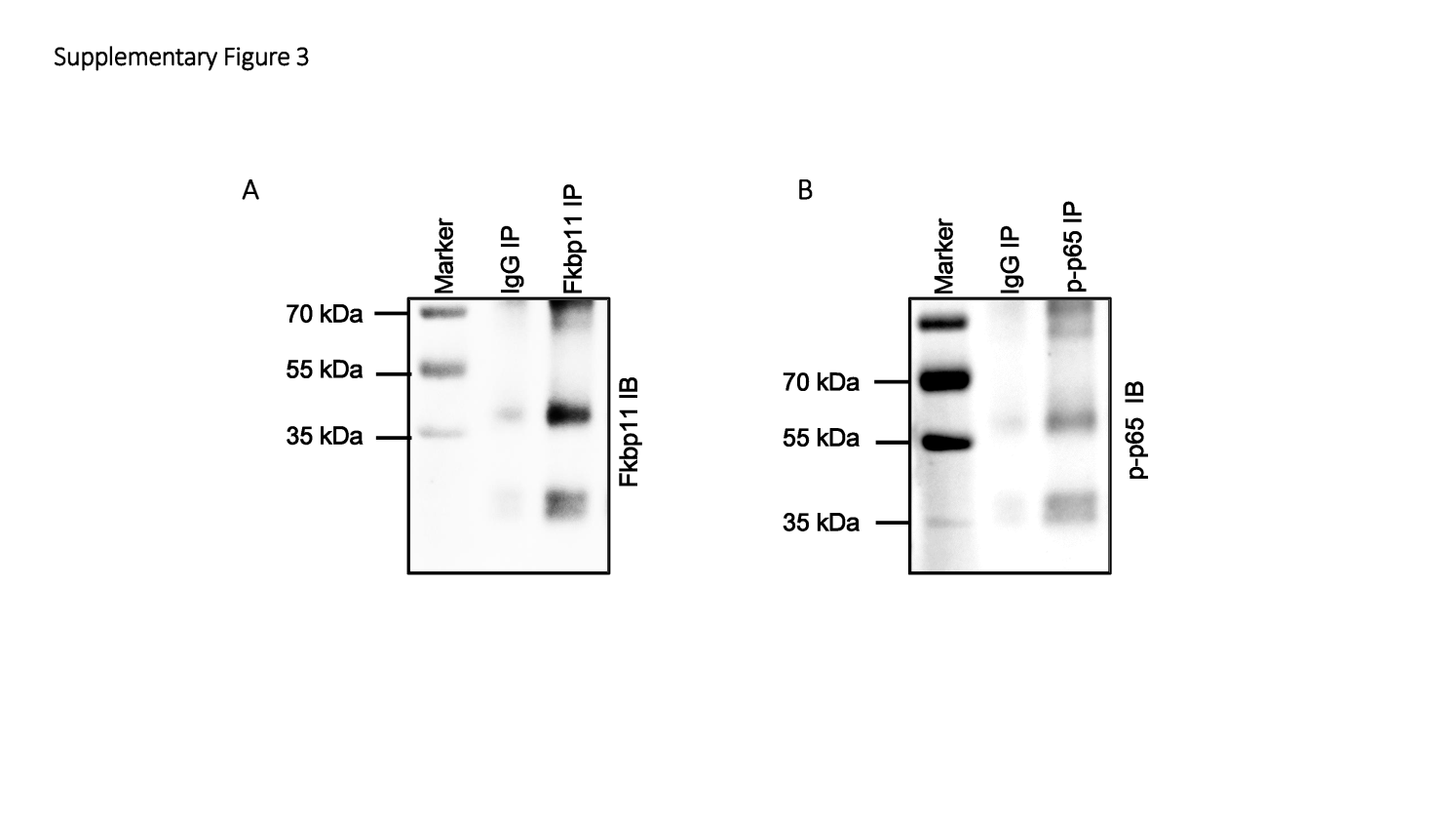


**Supplementary Figure 3.** **IgG controls for immunoprecipitation experiments (A)** Immunoblotting of Fkbp11 (Fkbp11 IB) following immunoprecipitation using antibodies against Fkbp11 or IgG control (IgG IP) from whole cell lysates of control EA.hy926 cells. **(B)** Immunoblotting of p-p65 (p-p65 IB) following immunoprecipitation using antibodies against p-p65 or IgG control (IgG IP) from whole cell lysates of control EA.hy926 cells.

# Supplementary Figure 4


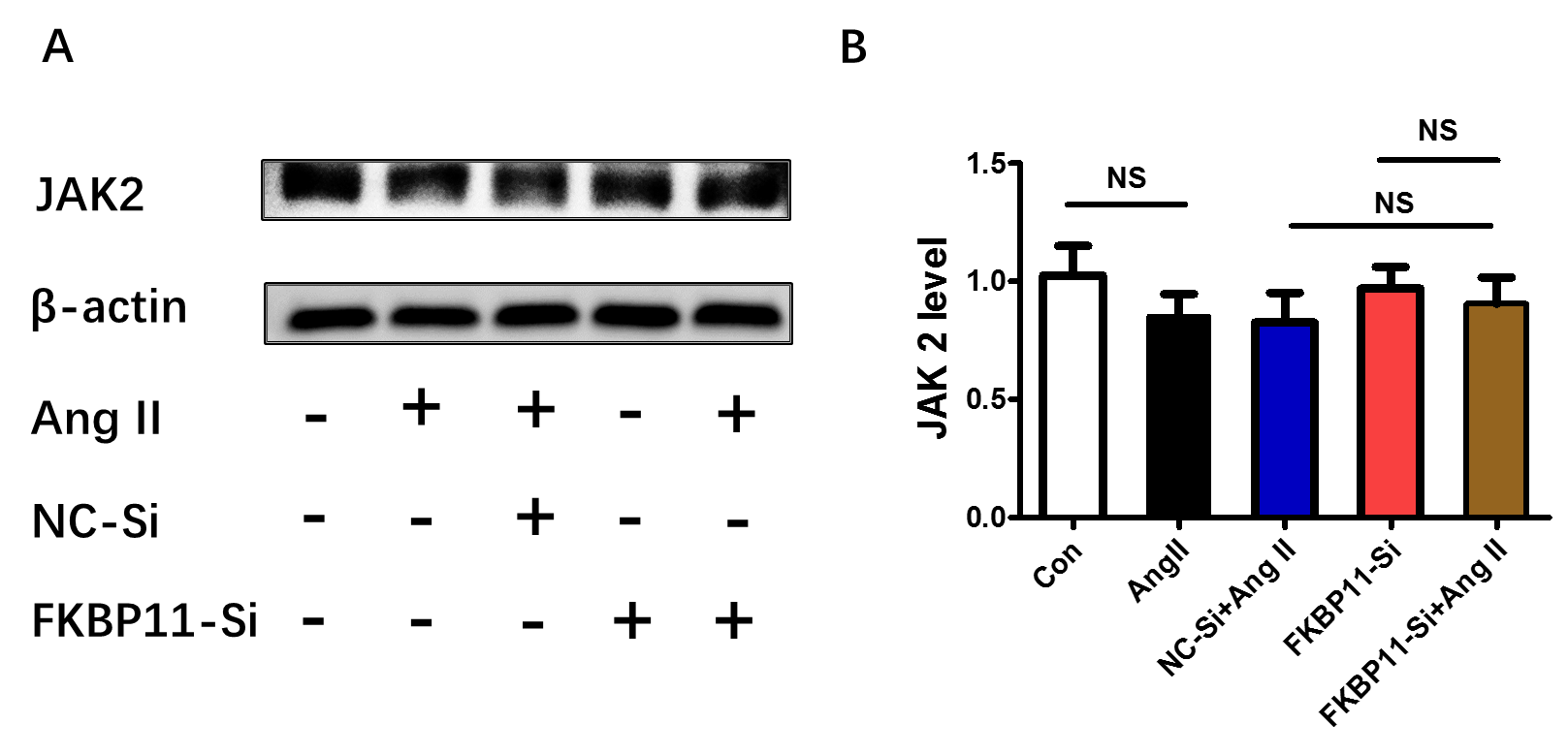


# Supplementary Figure 4. The role of FKBP11 in AAD was not linked to JAK2. Endothelial cells were incubated with Ang II after transfected with FKBP11-Si2 or scrambled control NC-Si. (A) Representative immunoblots and bar graph (B) showed FKBP11 siRNA treatment resulted in unaltered expression of JAK2 regardless of Ang II treatment CON: Control cells without treatment; Ang II: 1.0×10^-6^ mol/L Angiotensin II treated EA.hy926cells; NC-Si: Scrambled SiRNA for FKBP11; FKBP11-Si: FKBP11 knockdown SiRNA. Mean value ± SEM of at least three independent experiments (B); NS, not significant (ANOVA).

# Supplementary Figure 5

**
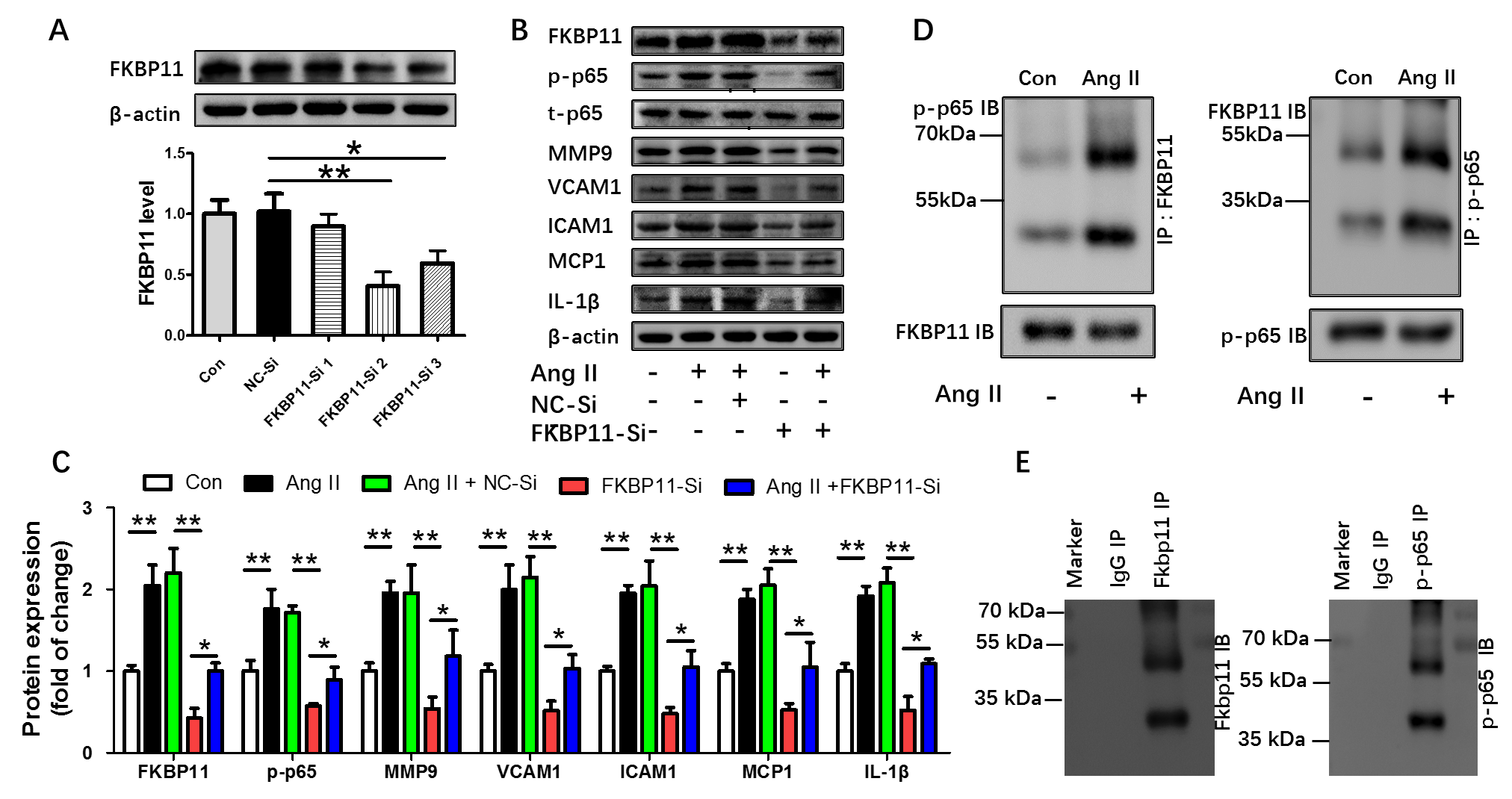
**

# Supplementary Figure 5. The pro-inflammatory function of FKBP11 and activation of NF-kB p65 subunit in primary endothelial cell HUVECs. (A) Representative immunoblots (upper panel) and bar graph (lower panel) showed FKBP11 specific siRNA FKBP11-Si2 and FKBP11-Si3 but not FKBP11-Si1 can effectively suppress the protein expression as compared to the scrambled control NC-Si in HUVECs. (B-D) HUVECs were incubated with Ang II after transfected with FKBP11-Si2 or scrambled control NC-Si. Representative immunoblots (B) and bar graph (C) showing FKBP11-siRNA treatment could effectively suppress the phosphorylation of p65 and subsequently the expression of pro-inflammatory cytokines MCP1, VCAM1, ICAM1 and IL1-β. (D) The interaction of FKBP11 and p-p65 was enhanced following Ang II treatment in HUVECs. Detection of p-p65 by immunoblotting following immunoprecipitation of FKBP11 from whole cell lysates of control (Con) cells or of cells after Ang II treatment (Ang II), left panel or vice versa, right panel; representative images of precipitated protein immunoblots (top) and respective input immunoblots (bottom). IgG controls for immunoprecipitation experiments were shown (E). CON: Control cells without treatment; Ang II: 1.0×10^-6^ mol/L Angiotensin II treated cells; NC-Si: Scrambled SiRNA for FKBP11; FKBP11-Si: FKBP11 knockdown SiRNA. Mean value ± SEM of at least three independent experiments (A, C); 🞳: *P* < 0.05; 🞳🞳: *P* < 0.01 (ANOVA); Representative immunoblots of at least three independent experiments (A, B, D, E); IB: immunoblot; IP: immunoprecipitation.
